# Negative Influence of Motor Impairments on Upper Limb Movement Patterns in Children with Unilateral Cerebral Palsy. A Statistical Parametric Mapping Study

**DOI:** 10.3389/fnhum.2017.00482

**Published:** 2017-10-05

**Authors:** Cristina Simon-Martinez, Ellen Jaspers, Lisa Mailleux, Kaat Desloovere, Jos Vanrenterghem, Els Ortibus, Guy Molenaers, Hilde Feys, Katrijn Klingels

**Affiliations:** ^1^Department of Rehabilitation Sciences, KU Leuven, Leuven, Belgium; ^2^Neural Control of Movement Lab, ETH Zurich, Zurich, Switzerland; ^3^Clinical Motion Analysis Laboratory, University Hospitals Leuven, Leuven, Belgium; ^4^Department of Development and Regeneration, KU Leuven, Leuven, Belgium; ^5^Department of Orthopedic Medicine, University Hospitals Leuven, Leuven, Belgium; ^6^Rehabilitation Research Center (REVAL), BIOMED, University of Hasselt, Diepenbeek, Belgium

**Keywords:** cerebral palsy, motor impairments, spasticity, muscle weakness, motion analysis, upper limb, neurorehabilitation, Statistical Parametric Mapping

## Abstract

Upper limb three-dimensional movement analysis (UL-3DMA) offers a reliable and valid tool to evaluate movement patterns in children with unilateral cerebral palsy (uCP). However, it remains unknown to what extent the underlying motor impairments explain deviant movement patterns. Such understanding is key to develop efficient rehabilitation programs. Although UL-3DMA has been shown to be a useful tool to assess movement patterns, it results in a multitude of data, challenging the clinical interpretation and consequently its implementation. UL-3DMA reports are often reduced to summary metrics, such as average or peak values per joint. However, these metrics do not take into account the continuous nature of the data or the interdependency between UL joints, and do not provide phase-specific information of the movement pattern. Moreover, summary metrics may not be sensitive enough to estimate the impact of motor impairments. Recently, Statistical Parametric Mapping (SPM) was proposed to overcome these problems. We collected UL-3DMA of 60 children with uCP and 60 typically developing children during eight functional tasks and evaluated the impact of spasticity and muscle weakness on UL movement patterns. SPM vector field analysis was used to analyze movement patterns at the level of five joints (wrist, elbow, shoulder, scapula, and trunk). Children with uCP showed deviant movement patterns in all joints during a large percentage of the movement cycle. Spasticity and muscle weakness negatively impacted on UL movement patterns during all tasks, which resulted in increased wrist flexion, elbow pronation and flexion, increased shoulder external rotation, decreased shoulder elevation with a preference for movement in the frontal plane and increased trunk internal rotation. Scapular position was altered during movement initiation, although scapular movements were not affected by muscle weakness or spasticity. In conclusion, we identified pathological movement patterns in children with uCP and additionally mapped the negative impact of spasticity and muscle weakness on these movement patterns, providing useful insights that will contribute to treatment planning. Last, we also identified a subset of the most relevant tasks for studying UL movements in children with uCP, which will facilitate the interpretation of UL-3DMA data and undoubtedly contribute to its clinical implementation.

## Introduction

An efficient use of the upper limb (UL) requires a fine-tuned coordination between head, trunk, arm and hand movements. This fine-tuned coordination is commonly impaired in children with unilateral cerebral palsy (uCP). They present with various motor and sensory impairments on one side of the body (Uvebrant, 1988), caused by a lesion in the developing brain (Bax et al., 2005). As a result, children with uCP often experience difficulties during various activities of daily life, ranging from simple reaching or grasping tasks to more complex movements such as object manipulation (Klingels et al., 2012). A vast body of literature has contributed to our understanding of the relation between motor and sensory impairments and UL activity limitations in children with uCP. For example, spasticity and muscle weakness at the level of the elbow and wrist have a negative impact on unimanual and bimanual task performance (Klingels et al., 2012; Brændvik et al., 2013). However, studies thus far mostly used clinical scales to assess UL function, which do not provide detailed quantitative information and, as such, lack the sensitivity to measure the fine-tuned coordination of UL function.

Apart from the clinical scales, three dimensional motion analysis (3DMA) offers a reliable and valid tool to examine UL movement patterns and coordination between the different joints (Jaspers et al., 2010). However, its output is complex due to the large amount of degrees of freedom involved in the UL, and the variety of tasks that can be measured. As a result, studies employing UL-3DMA mostly focus on temporal aspects of movement coordination during reaching (Chang et al., 2005; Butler and Rose, 2012), or report extracted metrics of joint angle kinematics such as maximum or minimum angle, range of motion, or end-point angles (Jaspers et al., 2011a). Based on these metrics, the negative impact of spasticity on trajectory straightness, peak velocity, or the number of movement units has already been demonstrated (Chang et al., 2005; van der Heide et al., 2005; Aboelnasr et al., 2017). Thus far, only two studies reported the negative relation between UL movement deviations, expressed as a summary index, and both muscle weakness and spasticity during various tasks (Jaspers et al., 2011c; Mailleux et al., 2017). Whilst these studies provide some first insights regarding the relation between motor deficits and UL movement pathology in uCP, results are based on an a-priori selection of extracted data points without a specific hypothesis, introducing bias in the results (Pataky, 2010, 2012; Pataky et al., 2016b).

Extracting specific data points, for example where the differences are maximum, leads to results that may exceed a certain α level that is uncorrected and unrepresentative for the actual number of data points in the dataset, which in turn increases the chances of committing a type I error (false positive). This “regional focus bias” questions the validity of currently used statistical inferences in 3DMA (Pataky et al., 2013). Recently, Statistical Parametric Mapping (SPM) has been proposed as a valid method to overcome the issues of multiple comparison, uncorrected threshold and interdependency between joint angles (vector components). SPM was originally developed for neuroimaging data, and has been transferred to the field of biomechanics to study bounded and continuous data. This analysis allows hypothesis testing over the entire waveform (Friston et al., 1991, 2007; Pataky, 2010) and reduces the chances of incorrectly rejecting the null hypothesis, since the number of statistical tests is lower (Pataky et al., 2013). However, the potential merit of SPM to investigate UL movement patterns has not yet been explored, which could offer valuable new insights that will help to further define a tailor-made UL treatment planning based on the individual needs of the child with uCP.

In this study, we used SPM for the first time to comprehensively assess UL movement patterns in children with uCP and in typically developing children (TDC). We first explored differences between both groups during eight tasks (reaching, reach-to-grasp, and gross motor tasks) and identified pronounced differences at all joint levels. Second, we investigated to what extent spasticity and muscle weakness at the level of the elbow and wrist impact on UL movement patterns in children with uCP, and found a negative influence of distal motor impairments at all joints except for the scapula. Finally, and based on these analyses, we aimed to identify the most discriminative and sensitive set of tasks to investigate UL movement patterns in children with uCP and proposed a selection of three tasks.

## Materials and methods

### Participants

This study included a cohort of 120 children, aged 5–15 years (60 spastic uCP, 60 TDC). Children with uCP were recruited via the CP care program of the University Hospital Leuven (Belgium) between 2010 and 2016. They were eligible to partake in the study if they were able to comprehend the test procedure and had sufficient UL function to actively open their hand. Children with uCP were excluded in case of previous UL surgery or botulinum neurotoxin-A injections 6 months prior to testing. TDC were recruited via schools and youth movements and were excluded in case of a history of any neurological or musculoskeletal disorder or previous UL surgery. This study was carried out in accordance with the recommendations of Ethical Committee of the University Hospital Leuven with written informed consent from all subjects. All subjects gave their verbal assent to participate and parents gave written informed consent in accordance with the Declaration of Helsinki. The protocol was approved by the Ethical Committee of the University Hospital Leuven (S55555; S56513).

### Procedure

All children underwent an UL-3DMA at the Clinical Motion Analysis Laboratory of the University Hospital Leuven (Belgium). Children with uCP additionally received a clinical UL evaluation at body function level, including an assessment of muscle weakness and spasticity, evaluated with the Manual Muscle Testing (Hislop and Montgomery, 2007), and the Modified Ashworth Scale (MAS) (Bohannon and Smith, 1987), respectively. The clinical evaluation of muscle tone and strength has been shown to be reliable in children with uCP (Klingels et al., 2010). Muscle weakness was evaluated for three muscle groups, i.e., elbow extensors, elbow supinators, and wrist extensors (total score: 0–15). Spasticity was assessed for three muscle groups, i.e., elbow flexors, elbow pronators, and wrist flexors (total score: 0–12). We opted for a composite score of these muscle groups based on a previous study (Klingels et al., 2012). All UL evaluations were conducted by four experienced physiotherapists (CSM, EJ, LM, CH).

UL-3DMA was conducted in a sitting position utilizing a custom-made chair that ensured foot and back-support. Seventeen reflective markers (14 mm diameter) were mounted on the trunk, acromion, upper and lower arm, and the hand, and several static calibration trials were performed to identify the anatomical landmarks of interest (Wu et al., 2005). The UL movement protocol consisted of eight tasks: three reaching tasks in different directions (forwards, RF; upwards, RU; sideways, RS), two reach-to-grasp tasks with different objects (grasp a sphere, RGS; or grasp a vertical cylinder, RGV), and three gross motor tasks simulating daily life activities (hand-to-head, HTH; hand-to-mouth, HTM; hand-to-shoulder, HTS). Reach and reach-to-grasp tasks were executed at shoulder height, except RU which was performed at eye height. All tasks were performed with the non-dominant/affected arm at self-selected speed. Children were instructed to repeat each task four times within one movement recording, two recordings were acquired per task, resulting in a total of eight movement repetitions per task. The starting position of every task was upright sitting with 90° of hip and knee flexion, hand on the ipsilateral knee. This protocol was shown to be reliable in both TDC and children with uCP (Jaspers et al., 2010, 2011b). For further details about the kinematic model, standardization and marker placement see Jaspers et al (Jaspers et al., 2010). Motion was recorded with 15 Vicon infrared cameras (Oxford Metrics, Oxford, UK) sampling at 100 Hz.

### Data processing

Data of 3D marker coordinates was processed offline using Vicon Nexus 1.8.5 software (Oxford Metrics, Oxford, UK). This data was filtered using a Woltring filtering routine with a predicted mean squared error of 10 mm^2^ (Woltring, 1995). Movement cycles with marker occlusion exceeding 20% of movement duration were excluded. If marker occlusion was <20%, spline interpolation gap filling, implemented in Nexus, was applied to the marker 3D coordinate data. Start (hand on ipsilateral knee) and end of each movement cycle were identified. Task end-point was defined as follows: (1) touch a sphere with the palm of the hand (RF, RU, and RS), (2) grasp an object [sphere (RGS) or vertical cylinder (RGV)], and (3) touch different parts of the body [top of the head (HTH), mouth (HTM), or contralateral shoulder (HTS)]. The first and last repetitions of each recording were excluded to avoid start and stop strategies of the child, resulting in a total of four movement cycles per task. Movement cycles were time normalized (0–100%) and the root mean squared error (RMSE) of the kinematic signals of each cycle was computed and compared to the mean of the remaining three (per task). The three cycles with lowest RMSE were utilized for further statistical analysis, to maximize repeatability in performance. UL kinematic calculations were computed with ULEMA v1.1.9 (MATLAB-based open source software, available for download at https://github.com/u0078867/ulema-ul-analyzer). Extracted UL kinematics consisted of five joints with a total of 12 angles: trunk [three degrees of freedom (DoF): rotation, lateral flexion, and flexion-extension], scapula (three DoF: tilting, pro-retraction, and rotation), shoulder (three DoF: rotation, elevation plane, and elevation), elbow (two DoF: flexion-extension and pro-supination), and wrist (one DoF: flexion-extension). The interpretation of joint angle kinematics can be found in the open source documentation of the ULEMA software (page 14, https://github.com/u0078867/ulema-ul-analyzer/blob/master/AppendicesI-II.pdf).

### Statistical analysis

Descriptive statistics were used to report demographic and clinical data. The normal distribution of age was verified in both groups using the Kolmogorov–Smirnov test (TDC, *p* = 0.20; uCP, *p* = 0.20) and age differences between groups were tested using an unpaired Student's *t*-test. We used chi-square test to compare gender frequency between groups. For the ordinal scorings of muscle tone and strength, median and interquartile ranges (IQR) were reported, and non-parametric statistics were computed.

SPM1d version 0.4 (MATLAB-based open source software, available for download at http://www.spm1d.org/) was used to conduct vector field analysis (joint level) and corresponding *post-hoc* analysis of each vector component (joint DoF) (Pataky, 2012). SPM1d is identical to the conventional inferential statistics, with the following differences: (1) it takes into account the covariance among the vector components (joint DoF), (2) it considers field smoothness and size when calculating the critical threshold (test statistic), and (3) it utilizes random field theory to compute probability of cluster-based threshold crossings (*p*-values). For every task, UL movement patterns were compared between groups (TDC vs. uCP) using the Hotelling's *T*^2^ test (SPM{T2}, analog to unpaired Student's *t*-test), with *post-hoc* scalar field *t*-tests for each vector component (SPM{t} per joint DoF). The relation between motor impairments and UL movement patterns in children with uCP was assessed using the non-parametrical Canonical Correlation analysis (SnPM{X2}, analog to linear regression), with *post-hoc* scalar field non-parametric linear regressions for each vector component (SnPM{t}, per joint DoF). Bonferroni correction was applied for *post-hoc* tests taking into account the number of components (DoF) of each vector (e.g., three components for the scapula: tilting, rotation, pro-retraction; two components for the elbow: pro-supination and flexion-extension).

**Figure 1:**
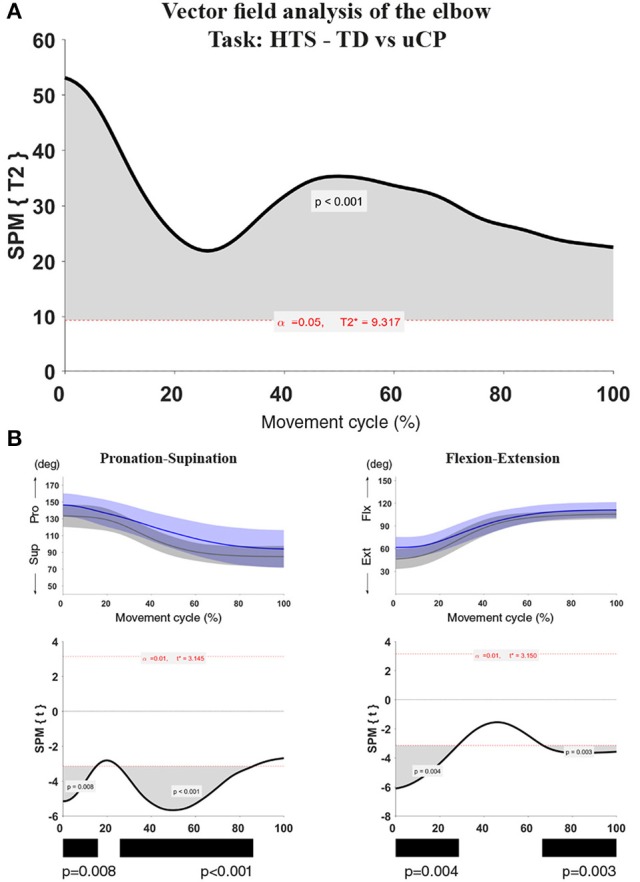
Statistical Parametric Mapping output example for the comparison of elbow kinematics (two vector components) during the HTS task between children with uCP and TDC. **(A)** Hoteling's test output with one cluster over 100% of the movement cycle, the bold black line is the computed t-curve, the dashed red line indicates the random theory threshold calculated for this test (at 9.317 for α < 0.05). Over 100% of the movement cycle, the vectors of the TDC and uCP group are significantly different; **(B)** Elbow vector decomposition (left: pro-supination; right: flexion-extension) with mean (bold line) and standard deviation (translucent area) of the TDC (gray) and uCP (blue) group. Below, the SPM{t} output correspondent to each of the vector components. Children with uCP show increased elbow flexion over 28 and 33% of the movement cycle in two different clusters (cluster 1: 0–28%, *p* < 0.01; cluster 2: 67–100%, *p* < 0.01) and increased pronation over 15 and 61% of the movement cycle in two different clusters (cluster 1: 0–15%, *p* < 0.01; cluster 2: 25–86%, *p* < 0.01). The black bars under each SPM{t} output correspond to the identified clusters. The SPM output will be represented in this summarized manner throughout the manuscript.

For each test, a statistical parametric map (SPM) was calculated by computing the conventional univariate statistic. Next, Random Field Theory was used to estimate (1) the critical threshold above which only 5% (i.e., α < 0.05) of equally smoothed random data would be expected to cross, and (2) the probability that this would occur (i.e., *p*-value). If an SPM{t} crosses the critical threshold, this was identified as a statistically significant cluster at the vector field level. In case significance was reached in the vector field analysis, the correspondent *post-hoc* scalar field analysis was performed. When clusters were identified, information regarding the extent (percentage of the movement cycle), location (start and end points of the cluster), and a single *p*-value for each identified cluster was provided (see example in Figure 1).

## Results

### Participants

Sixty children with uCP [mean age (SD) = 10 y 3 m (2 y 4 m), 25 girls, 29 left hand affected] and 60 TDC [mean age (SD) = 10 y 2 m (3 y 1 m), 24 girls, 53 right handed (left UL assessed)] participated in the study (Table 1). Age was not significantly different between groups (*p* = 0.80). Chi-square test showed no differences in gender frequency between groups (*p* = 0.85). According to the Manual Ability Classification System (MACS, Eliasson et al., 2006), 18 children with uCP were classified as level I, 28 as level II, and 14 as level III. Muscle weakness median score was 10.5 (IQR = 1.6), and spasticity median score was 3.5 (IQR = 2.0). Eight children showed no spasticity in any muscle, and the remaining 52 children presented with spasticity in at least one of the three muscles (sum score > 1). One child did not have any muscle weakness, whereas all other children presented with muscle weakness in at least one of the three muscles (sum score < 15).

**Table 1:** Demographic and clinical characteristics of the study participants.

		**uCP (*n* = 60)**	**TDC (*n* = 60)**
Age	Mean, SD (range)	10y3m, 2y6m (5y2m–15y2m)	10y3m, 3y1m (5y–15y7m)
Gender	Boys [*n* (%)]	35 (58%)	36 (60%)
	Girls [*n* (%)]	25 (42%)	24 (40%)
Handedness (dominant or non-affected hand)	Right [*n* (%)]	29 (49%)	53 (88%)
	Left [*n* (%)]	31 (51%)	7 (12%)
MACS levels	I [*n* (%)]	18 (30%)	–
	II [*n* (%)]	28 (47%)	–
	III [*n* (%)]	14 (23%)	–
Muscle weakness^*^	Median (IQR)	10.5 (2.0)	–
Spasticity^**^	Median (IQR)	3.5 (2.0)	–

*uCP, unilateral cerebral palsy; TDC, typically developing children; MACS, manual ability classification system; IQR, interquartile range*.

*Age was not significantly different between groups (unpaired t-test, p = 0.80)*.

* *Muscle weakness for wrist and elbow extensors and elbow supinators (range 0–5 per muscle, total range 0–15)*.

** *Spasticity scores for wrist and elbow flexors and elbow pronators (range 0–4 per muscle, total range 0–12)*.

### UL movement patterns

In the following section, we will first report differences in UL movement patterns between children with uCP and TDC for each of the tasks. Next, we will describe the impact of muscle weakness and spasticity on UL movement patterns in children with uCP. Vector field analysis results are described with subsequent *post-hoc* analysis, if applicable. In general, results comprise a summary description of the identified clusters by SPM.

#### uCP vs. TDC

**Figure 2:**
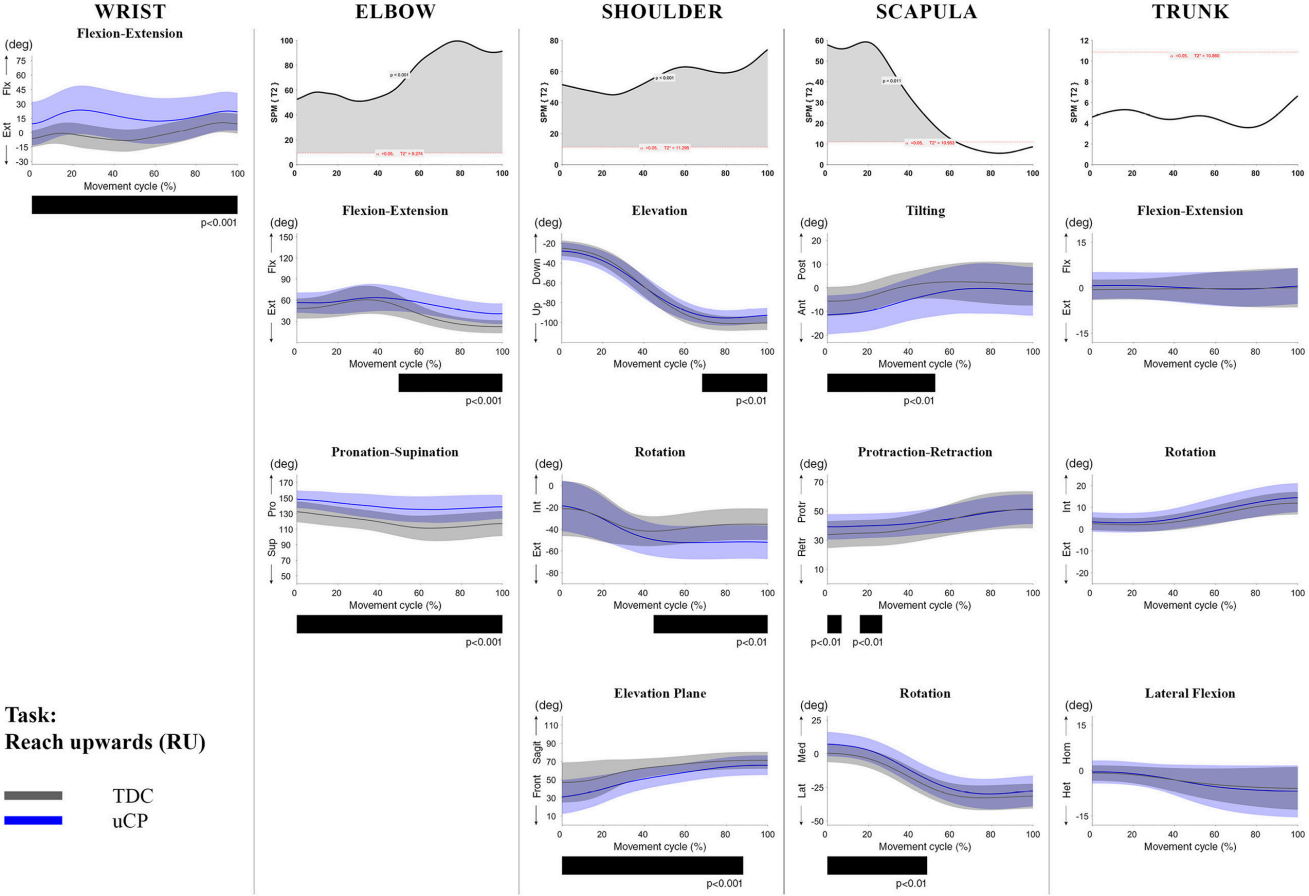
Comparison between children with uCP and TDC during the RU task for all joint angles. Each column corresponds to a joint (from left to right: wrist, elbow, shoulder, scapula, and trunk). The top image of each column is the SPM output of the vector field analysis (Hoteling's test, except for the wrist, where a *t*-test was computed). Below, mean (bold line) and standard deviation (translucent area) of the TDC (gray) and uCP (blue) of each vector component, and the respective *post-hoc* SPM{t} output. The black bar under each kinematic profile indicates clusters of significant difference between groups.

**Figure 3:**
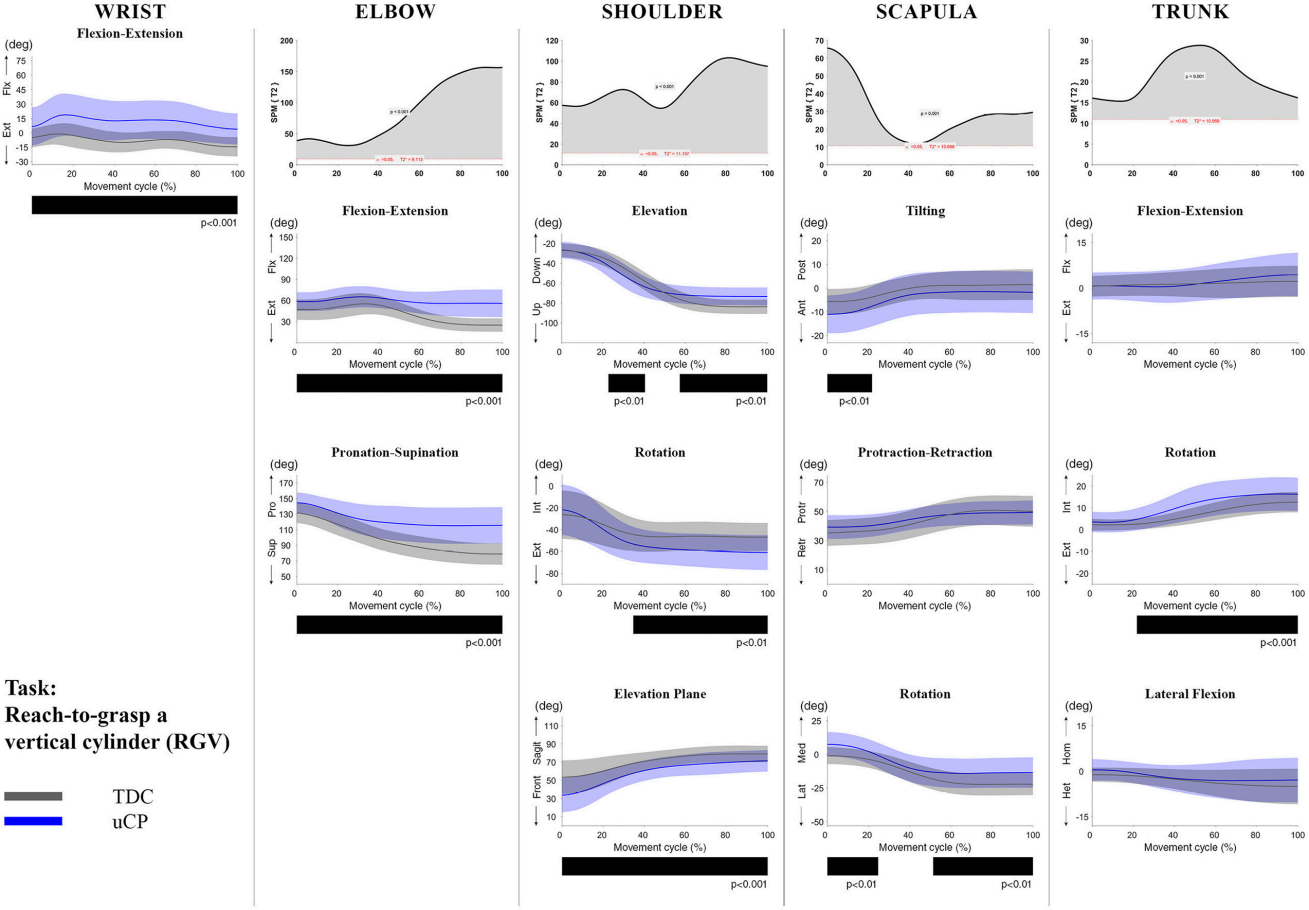
Comparison between children with uCP and TDC during the RGV task for all joint angles. Each column corresponds to a joint (from left to right: wrist, elbow, shoulder, scapula, and trunk). The top image of each column is the SPM output of the vector field analysis (Hoteling's test, except for the wrist, where a *t*-test was computed). Below, mean (bold line) and standard deviation (translucent area) of the TDC (gray) and uCP (blue) of each vector component, and the respective *post-hoc* SPM{t} output. The black bar under each kinematic profile indicates clusters of significant difference between groups.

**Figure 4:**
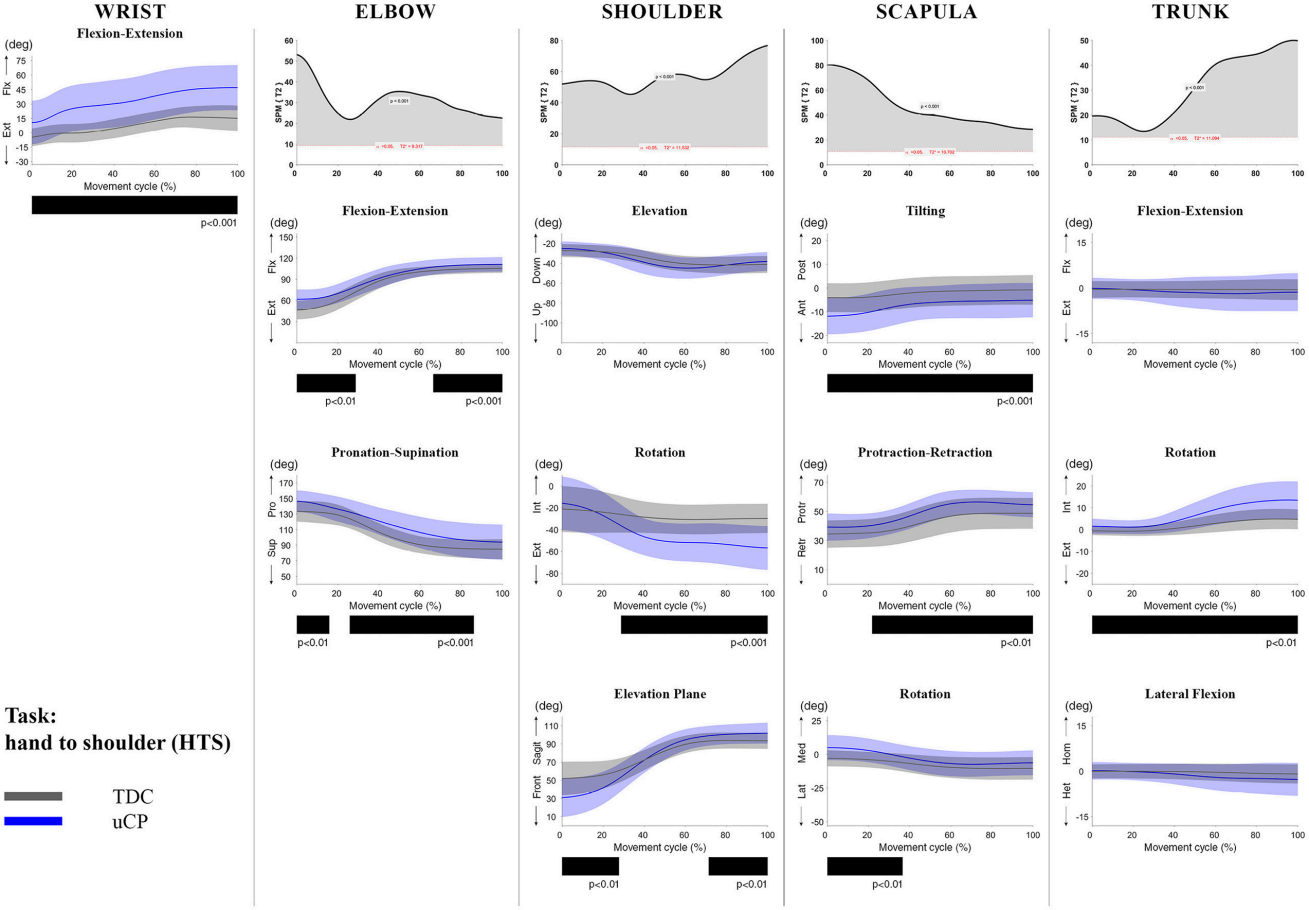
Comparison between children with uCP and TDC during the HTS task for all joint angles. Each column corresponds to a joint (from left to right: wrist, elbow, shoulder, scapula, and trunk). The top image of each column is the SPM output of the vector field analysis (Hoteling's test, except for the wrist, where a *t*-test was computed). Below, mean (bold line) and standard deviation (translucent area) of the TDC (gray) and uCP (blue) of each vector component, and the respective *post-hoc* SPM{t} output. The black bar under each kinematic profile indicates clusters of significant difference between groups.

Results regarding the comparison between uCP and TDC for all UL joints can be found in Table 2 (all tasks), Figures 2–4 (RU, RGV, HTS) and Supplementary Material 1 (RF, RS, RGS, HTM, HTH).

**Table 2:** Statistical Parametric Mapping comparison of movement patters in children with uCP and TDC.

			**RF**	**RU**	**RS**	**RGS**	**RGV**	**HTH**	**HTM**	**HTS**
WRIST	Flexion Extension	*n* clusters (*p*-value)	1 (<0.0001)	1 (<0.0001)	1 (<0.0001)	1 (<0.0001)	1 (<0.0001)	1 (<0.0001)	1 (<0.0001)	1 (<0.0001)
		% curve (range)	100% (0–100)	100% (0–100)	100% (0–100)	100% (0–100)	100% (0–100)	80% (0–80)	100% (0–100)	100% (0–100)
ELBOW	VECTOR FIELD	*n* clusters (*p*-value)	1 (<0.0001)	1 (<0.0001)	1 (<0.0001)	1 (<0.0001)	1 (<0.0001)	1 (<0.0001)	1 (<0.0001)	1 (<0.0001)
		% curve (range)	100% (0–100)	100% (0–100)	100% (0–100)	100% (0–100)	100% (0–100)	100% (0–100)	100% (0–100)	100% (0–100)
	Flexion Extension	*n* clusters (*p*-value)	2 (<0.01; <0.001)	1 (<0.001)	1 (<0.001)	2 (<0.01; <0.0001)	1 (<0.0001)	1 (<0.01)	1 (<0.01)	2 (<0.01; <0.01)
		% curve (range)	12% 52% (0–12) (48–100)	51% (49–100)	50% (50–100)	14% 64% (0–14) (36–100)	100% (0–100)	15% (0–15)	30% (0–30)	28% 33% (0–28) (67–100)
	Pro-supination	*n* clusters (*p*-value)	1 (<0.0001)	1 (<0.0001)	1 (<0.0001)	1 (<0.0001)	1 (<0.0001)	1 (<0.0001)	1 (<0.0001)	2 (<0.01; <0.001)
		% curve (range)	100% (0–100)	100% (0–100)	100% (0–100)	100% (0–100)	100% (0–100)	100% (0–100)	100% (0–100)	15% 61% (0–15) (25–86)
SHOULDER	VECTOR FIELD	*n* clusters (*p*-value)	1 (<0.0001)	1 (<0.0001)	1 (<0.0001)	1 (<0.0001)	1 (<0.0001)	1 (<0.0001)	1 (<0.0001)	1 (<0.0001)
		% curve (range)	100% (0–100)	100% (0–100)	100% (0–100)	100% (0–100)	100% (0–100)	100% (0–100)	100% (0–100)	100% (0–100)
	Elevation	*n* clusters (*p*-value)	1 (<0.01)	1 (<0.01)	–	2 (<0.01; <0.01)	2 (<0.01; <0.01)	1 (<0.01)	1 (<0.01)	–
		% curve (range)	19% (81–100)	31% (69–100)	–	28% 35% (17–45) (65–100)	18% 43% (22–40) (57–100)	24% (76–100)	5% (0–5)	–
	Rotation	*n* clusters (*p*-value)	1 (<0.01)	1 (<0.01)	1 (<0.01)	1 (<0.01)	1 (<0.01)	1 (<0.001)	1 (<0.01)	1 (<0.001)
		% curve (range)	50% (50–100)	55% (45–100)	77% (23–100)	59% (41–100)	66% (34–100)	74% (26–100)	67% (33–100)	72% (28–100)
	Elevation plane	*n* clusters (*p*-value)	1 (<0.0001)	1 (<0.0001)	1 (<0.01)	1 (<0.0001)	1 (<0.0001)	1 (<0.01)	1 (<0.01)	2 (<0.01; <0.01)
		% curve (range)	100% (0–100)	88% (0–88)	7% (0–7)	100% (0–100)	100% (0–100)	41% (0–41)	38% (0–38)	28% 28% (0–28) (72–100)
SCAPULA	VECTOR FIELD	*n* clusters (*p*-value)	1 (<0.001)	1 (<0.01)	1 (0.013)	2 (0.017; 0.026)	1 (<0.01)	1 (0.023)	1 (<0.001)	1 (<0.001)
		% curve (range)	100% (0–100)	63% (0–63)	62% (0–62)	54% 42% (0–54) (58–100)	100% (0–100)	41% (0–41)	100% (0–100)	100% (0–100)
	Tilting	*n* clusters (*p*-value)	1 (<0.01)	1 (<0.01)	1 (<0.01)	1 (<0.01)	1 (<0.01)	1 (<0.01)	1 (<0.01)	1 (<0.0001)
		% curve (range)	59% (0–59)	52% (0–52)	51% (0–51)	53% (0–53)	22% (0–22)	39% (0–39)	44% (0–44)	100% (0–100)
	Pro-retraction	*n* clusters (*p*-value)	–	2 (<0.01; <0.01)	1 (<0.01)	1 (<0.01)	–	–	–	1 (<0.01)
		% curve (range)	–	6% 11% (0–6) (15–26)	47% (12–59)	11% (0–11)	–	–	–	79% (21–100)
	Rotation	*n* clusters (*p*-value)	2 (<0.01; <0.01)	1 (<0.01)	1 (<0.01)	1 (<0.01)	2 (<0.01; <0.01)	1 (<0.01)	1 (<0.01)	1 (<0.01)
		% curve (range)	49% 10% (0–49) (90–100)	48% (0–48)	20% (0–20)	20% (0–20)	25% 48% (0–25) (52–100)	24% (0–24)	77% (0–77)	37% (0–37)
TRUNK	VECTOR FIELD	*n* clusters (*p*-value)	0	0	2 (0.046; 0.035)	1 (0.049)	1 (<0.001)	1 (<0.01)	1 (<0.01)	1 (<0.001)
		% curve (range)	–	–	15% 33% (1–15) (67–100)	6% (48–54)	100% (0–100)	66% (33–100)	100% (0–100)	100% (0–100)
	Axial Rotation	*n* clusters (*p*-value)	–	–	1 (<0.01)	–	1 (<0.001)	1 (<0.001)	1 (<0.0001)	1 (<0.0001)
		% curve (range)	–	–	32% (68–100)	–	78% (22–100)	66% (34–100)	100% (0–100)	100% (0–100)

*uCP, unilateral cerebral palsy; TDC, typically developing children; RF, reach forwards; RU, reach upwards; RS, reach sideways; RGS, reach-to-grasp a sphere; RGV, reach-to-grasp a vertical cylinder; HTH, hand-to-head; HTM, hand-to-mouth; HTS, hand-to-shoulder; n clusters, number of identified clusters; p-value, probability of the identified cluster: α < 0.05 vector field analysis, α < 0.017 (post-hoc with three components), α < 0.025 (post-hoc with two components); % curve, extent of the cluster over the waveform; range, start, and end point of the identified cluster*.

At the level of the **wrist** (one DoF), *t*-test comparison showed *increased wrist flexion* in the uCP group during 100% of the movement (all tasks, *p* < 0.001), except during HTH (first 80% of the movement cycle, *p* < 0.001).

Vector field analysis at the **elbow** (two DoF) showed significant kinematic differences between the two groups during the entire movement cycle (all tasks, 100%, *p* < 0.001). *Post-hoc t*-tests showed significantly *increased pronation* (all tasks, at least 75% of movement cycle, *p* < 0.01) and *elbow flexion* (all tasks, approx. between 15 and 100% of the movement cycle, *p* < 0.01) in uCP compared to TDC.

At the **shoulder** level (three DoF), we found significant differences between both groups during 100% of the movement cycle (all tasks, *p* < 0.001). Overall, *post-hoc t*-tests identified that shoulder movement patterns of children with uCP were characterized by increased *external rotation* (approx. between 30 and 100%, *p* < 0.01), increased *elevation in the frontal plane* (at least 40% of the movement cycle, *p* < 0.01) and *increased elevation* in movement initiation during the reach-to-grasp tasks (~0–40%, *p* < 0.01) that significantly decreased toward the end of the movement cycle (approx. between 60 and 100%, *p* < 0.01).

For the **scapula** (three DoF), SPM vector field analysis showed significant kinematic differences RF, RGV, HTM, and HTS (100%, *p* < 0.01), and during a large extent of the movement cycle for RGS (cluster 1: 0–54%, *p* < 0.02; cluster 2: 58–100%, *p* < 0.03), RS (0–80%, *p* = 0.01), RU (0–60%, *p* = 0.01), and HTH (0–40%, *p* = 0.02). Scapular kinematics of children with uCP were characterized by *increased anterior tilting* (all tasks, 0–40%, *p* < 0.01), *medial rotation* (all tasks during movement initiation, *p* < 0.01) and *protraction* (different locations of the movement cycle, *p* < 0.01).

**Trunk** kinematics (three DoF) were significantly different between the two groups of RGV, HTM, and HTS (100%, *p* < 0.003). Smaller differences were found for RGS (48–54%, *p* < 0.05), RS (cluster 1: 1–15%, *p* < 0.05; cluster 2: 67–100%, *p* = 0.03), and HTH (33–100%, *p* < 0.01). *Post-hoc* analysis showed that trunk kinematics of children with uCP were characterized by *increased inwards rotation* for RGV, HTM, and HTS tasks (*p* < 0.01) and *increased outwards rotation* at the end of the movement cycle for RS (*p* < 0.01).

#### The impact of motor impairments on UL movement patterns

**Figure 5:**
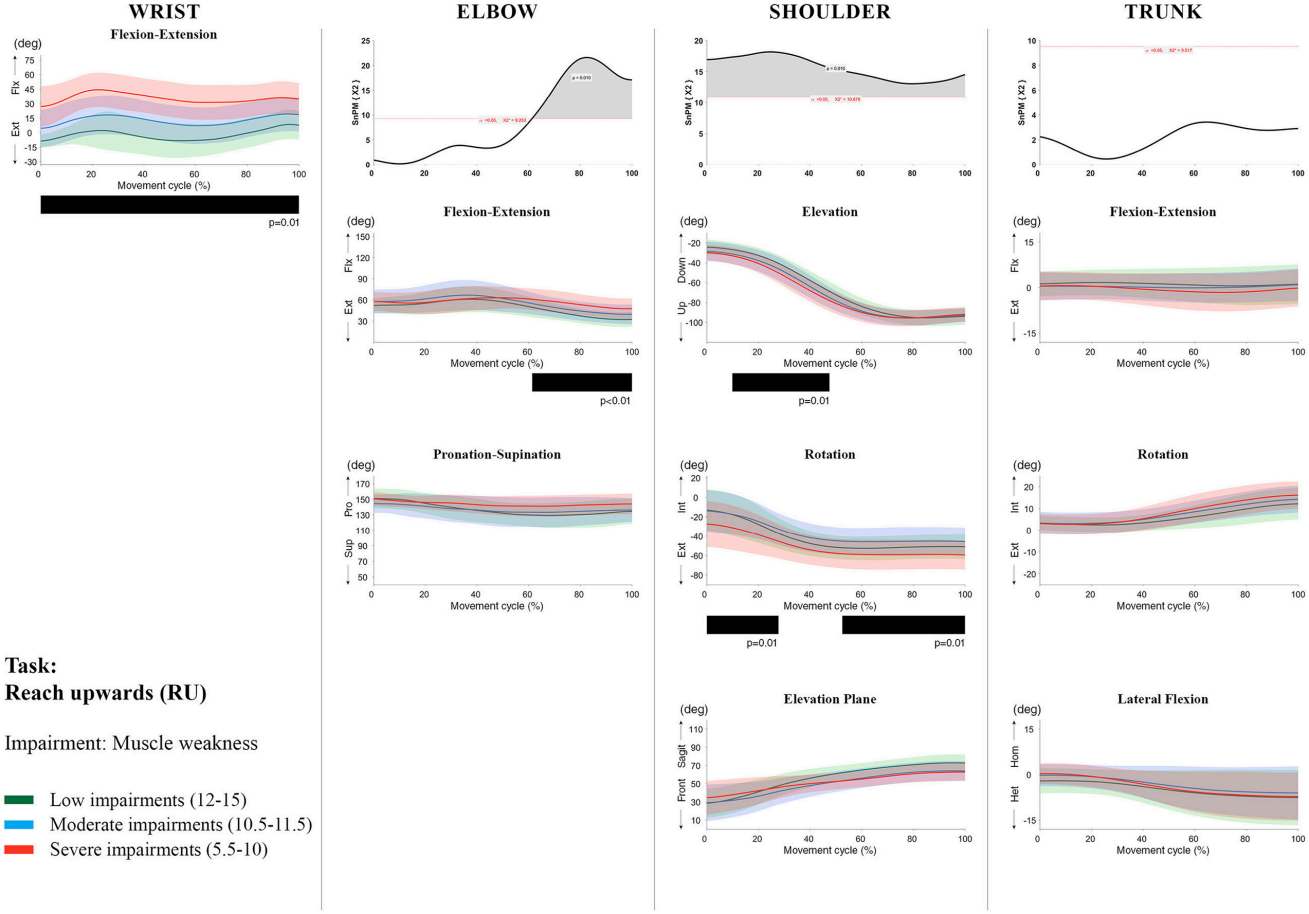
Impact of muscle weakness on UL movement patterns in children with uCP during the RU task for all joint angles. Each column corresponds to a joint (from left to right: wrist, elbow, shoulder, and trunk). The top image of each column is the SPM output of the vector field analysis (non-parametric Canonical Correlation Analysis test, except for the wrist, where a non-parametric linear regression was computed). For visualization purposes, kinematic data was grouped according to the level of motor impairments, i.e., muscle weakness total score: low impairments (score between 12 and 15, i.e., values above percentile 75), moderate impairments (score between 10.5 and 11.5, i.e., values between percentile 25 and 75), and severe impairments (score between 5.5 and 10, i.e., values below percentile 25). Below, mean (bold line) and standard deviation (translucent area) of the low impairments (green), moderate impairments (blue), and severe impairments (red) of each vector component and the respective *post-hoc* SPM{t} output. The black bar under each kinematic profile indicates clusters of significant influence of the impairment level on the kinematic variable.

**Figure 6:**
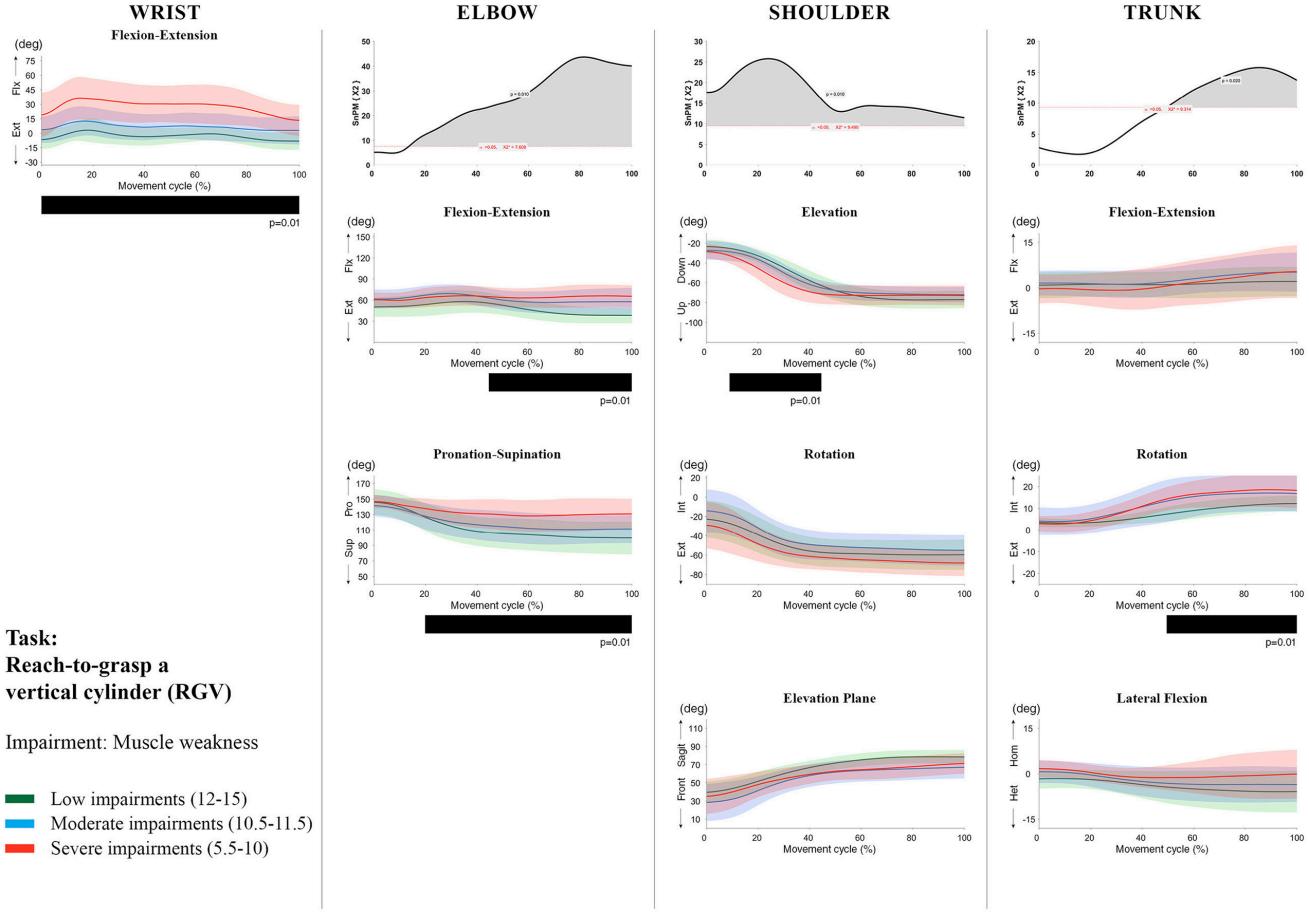
Impact of muscle weakness on UL movement patterns in children with uCP during the RGV task for all joint angles. Each column corresponds to a joint (from left to right: wrist, elbow, shoulder, and trunk). The top image of each column is the SPM output of the vector field analysis (non-parametric Canonical Correlation Analysis test, except for the wrist, where a non-parametric linear regression was computed). For visualization purposes, kinematic data was grouped according to the level of motor impairments, i.e., muscle weakness total score: low impairments (score between 12 and 15, i.e., values above percentile 75), moderate impairments (score between 10.5 and 11.5, i.e., values between percentile 25 and 75), and severe impairments (score between 5.5 and 10, i.e., values below percentile 25). Below, mean (bold line) and standard deviation (translucent area) of the low impairments (green), moderate impairments (blue), and severe impairments (red) of each vector component and the respective *post-hoc* SPM{t} output. The black bar under each kinematic profile indicates clusters of significant influence of the impairment level on the kinematic variable.

**Figure 7:**
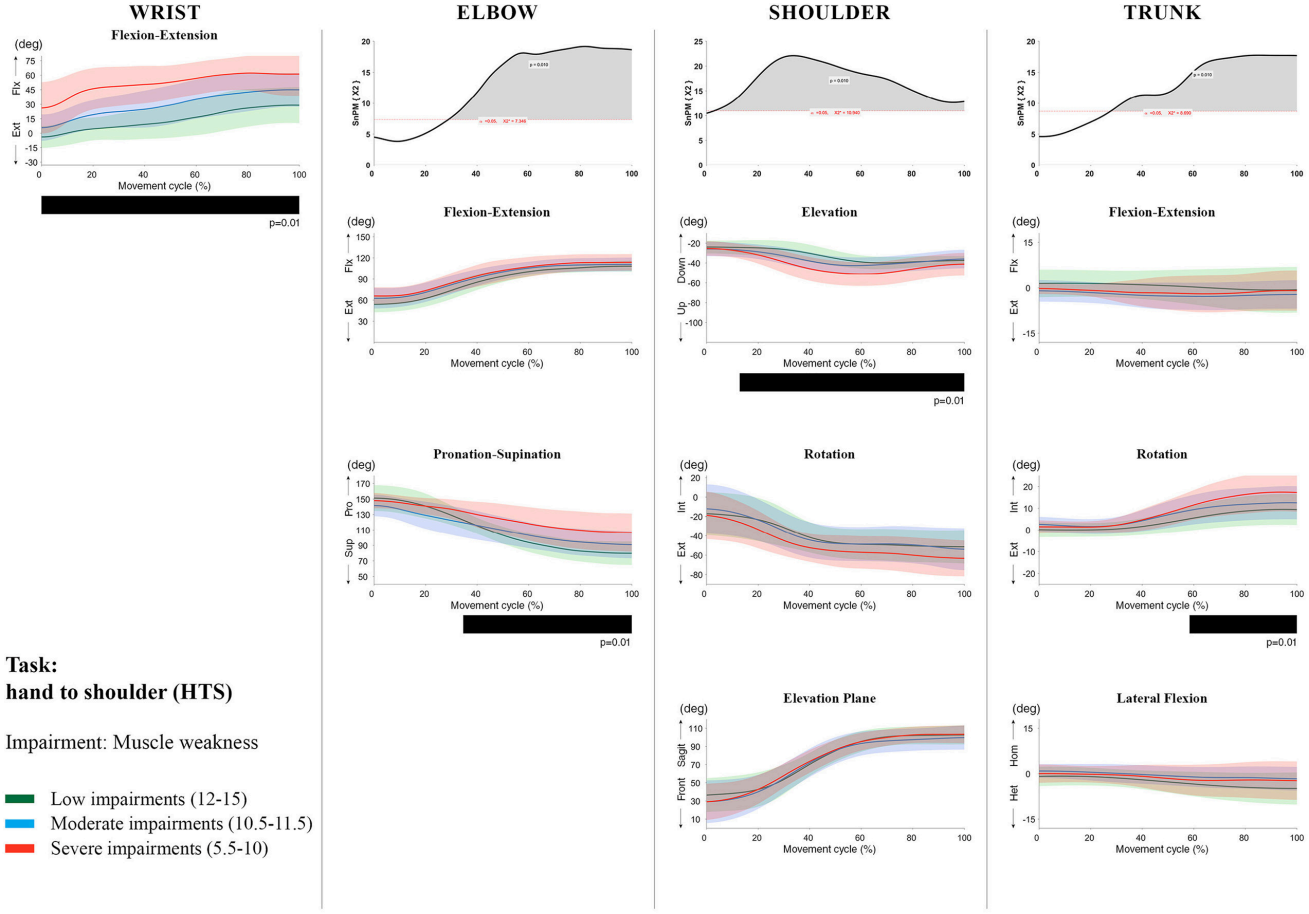
Impact of muscle weakness on UL movement patterns in children with uCP during the HTS task for all joint angles. Each column corresponds to a joint (from left to right: wrist, elbow, shoulder, and trunk). The top image of each column is the SPM output of the vector field analysis (non-parametric Canonical Correlation Analysis test, except for the wrist, where a non-parametric linear regression was computed). For visualization purposes, kinematic data was grouped according to the level of motor impairments, i.e., muscle weakness total score: low impairments (score between 12 and 15, i.e., values above percentile 75), moderate impairments (score between 10.5 and 11.5, i.e., values between percentile 25 and 75), and severe impairments (score between 5.5 and 10, i.e., values below percentile 25). Below, mean (bold line) and standard deviation (translucent area) of the low impairments (green), moderate impairments (blue), and severe impairments (red) of each vector component and the respective *post-hoc* SPM{t} output. The black bar under each kinematic profile indicates clusters of significant influence of the impairment level on the kinematic variable.

**Figure 8:**
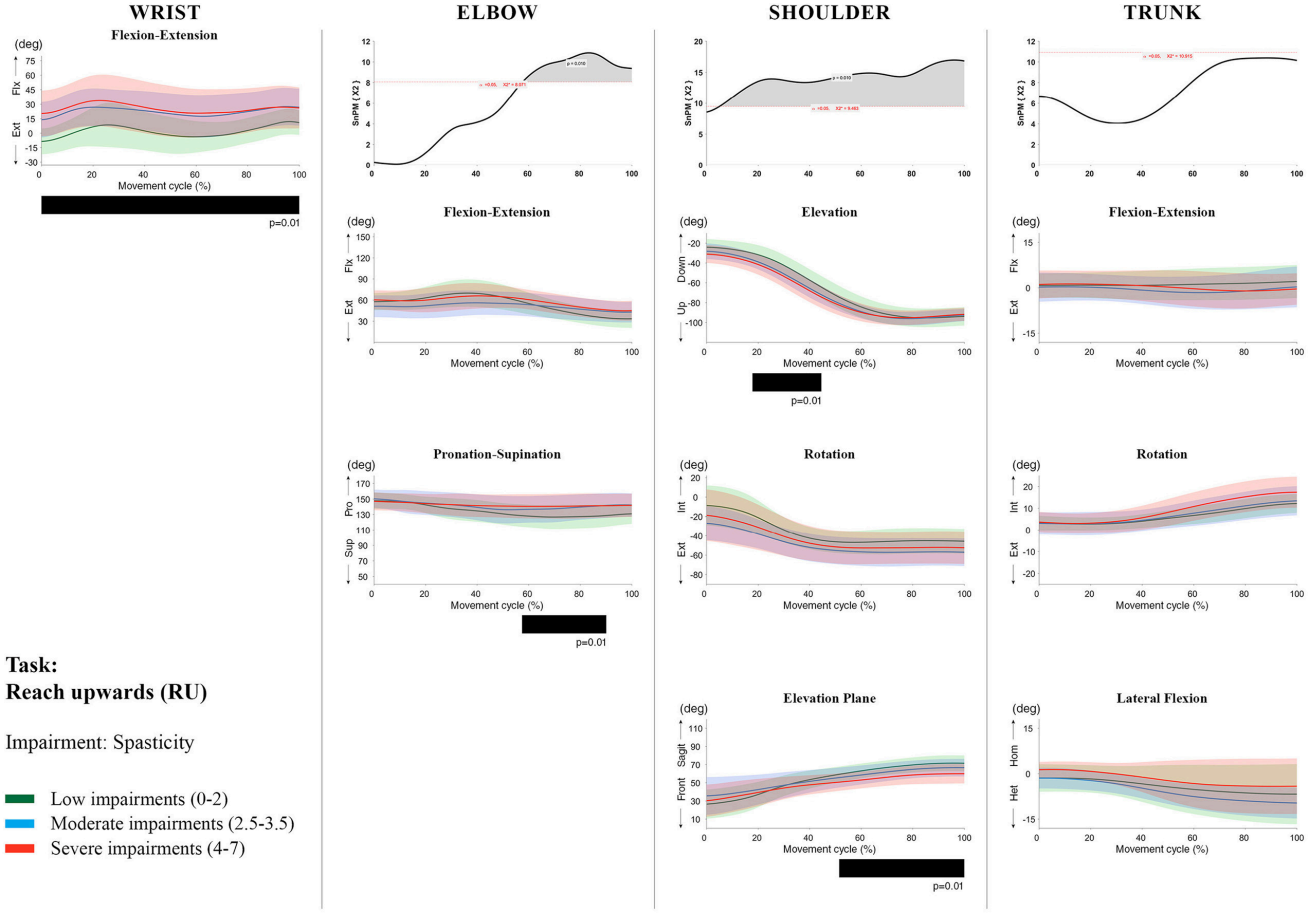
Impact of spasticity on UL movement patterns in children with uCP during the RU task for all joint angles. Each column corresponds to a joint (from left to right: wrist, elbow, shoulder, and trunk). The top image of each column is the SPM output of the vector field analysis (non-parametric Canonical Correlation Analysis test, except for the wrist, where a non-parametric linear regression was computed). For visualization purposes, kinematic data was grouped according to the level of motor impairments, i.e., spasticity total score: low impairments (score between 0 and 2, i.e., values above percentile 75), moderate impairments (score between 2.5 and 3.5, i.e., values between percentile 25 and 75), and severe impairments (score between 4 and 7, i.e., values below percentile 25). Below, mean (bold line) and standard deviation (translucent area) of the low impairments (green), moderate impairments (blue), and severe impairments (red) of each vector component and the respective *post-hoc* SPM{t} output. The black bar under each kinematic profile indicates clusters of significant influence of the impairment level on the kinematic variable.

**Figure 9:**
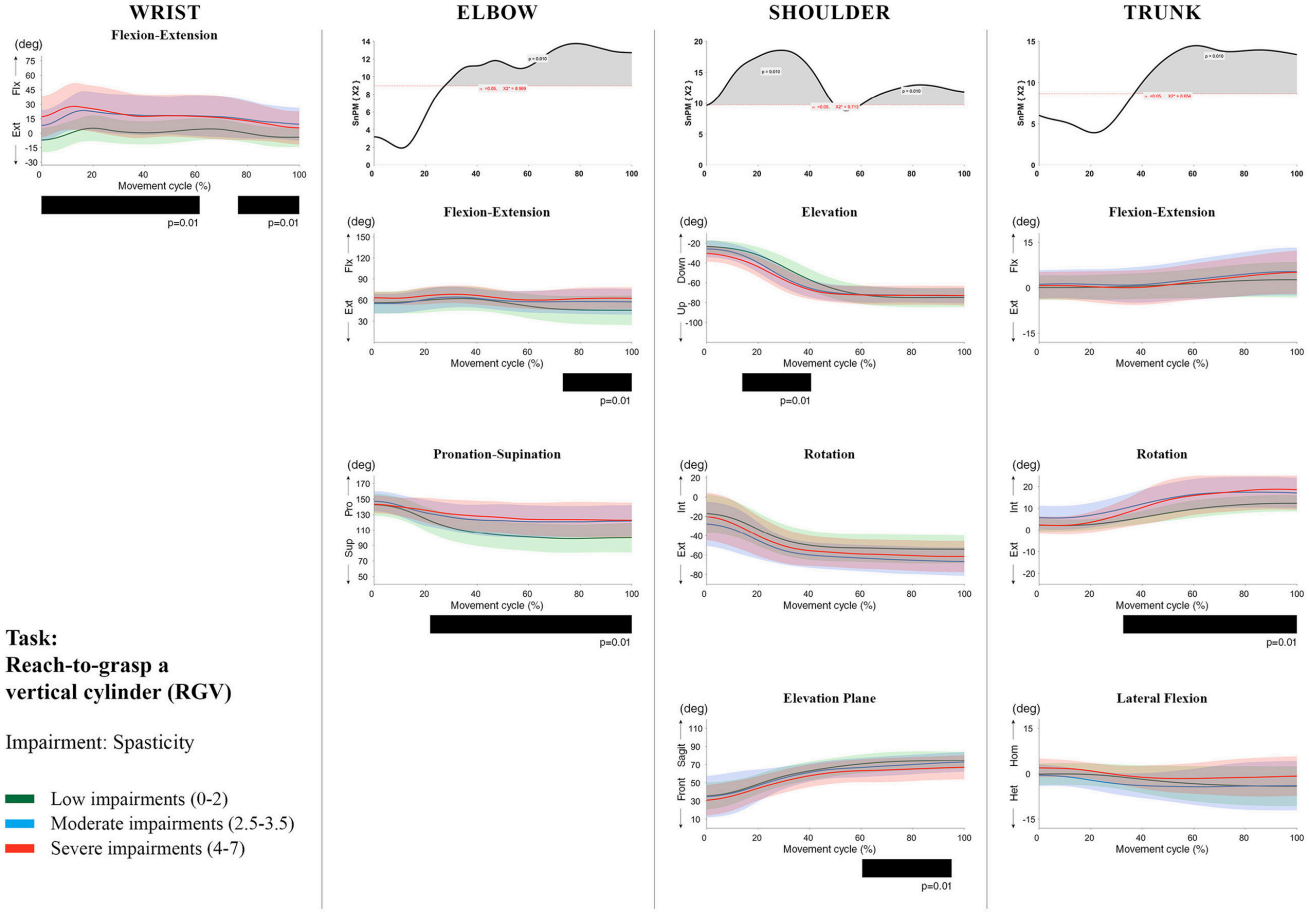
Impact of spasticity on UL movement patterns in children with uCP during the RGV task for all joint angles. Each column corresponds to a joint (from left to right: wrist, elbow, shoulder, and trunk). The top image of each column is the SPM output of the vector field analysis (non-parametric Canonical Correlation Analysis test, except for the wrist, where a non-parametric linear regression was computed). For visualization purposes, kinematic data was grouped according to the level of motor impairments, i.e., spasticity total score: low impairments (score between 0 and 2, i.e., values above percentile 75), moderate impairments (score between 2.5 and 3.5, i.e., values between percentile 25 and 75), and severe impairments (score between 4 and 7, i.e., values below percentile 25). Below, mean (bold line) and standard deviation (translucent area) of the low impairments (green), moderate impairments (blue), and severe impairments (red) of each vector component and the respective *post-hoc* SPM{t} output. The black bar under each kinematic profile indicates clusters of significant influence of the impairment level on the kinematic variable.

**Figure 10:**
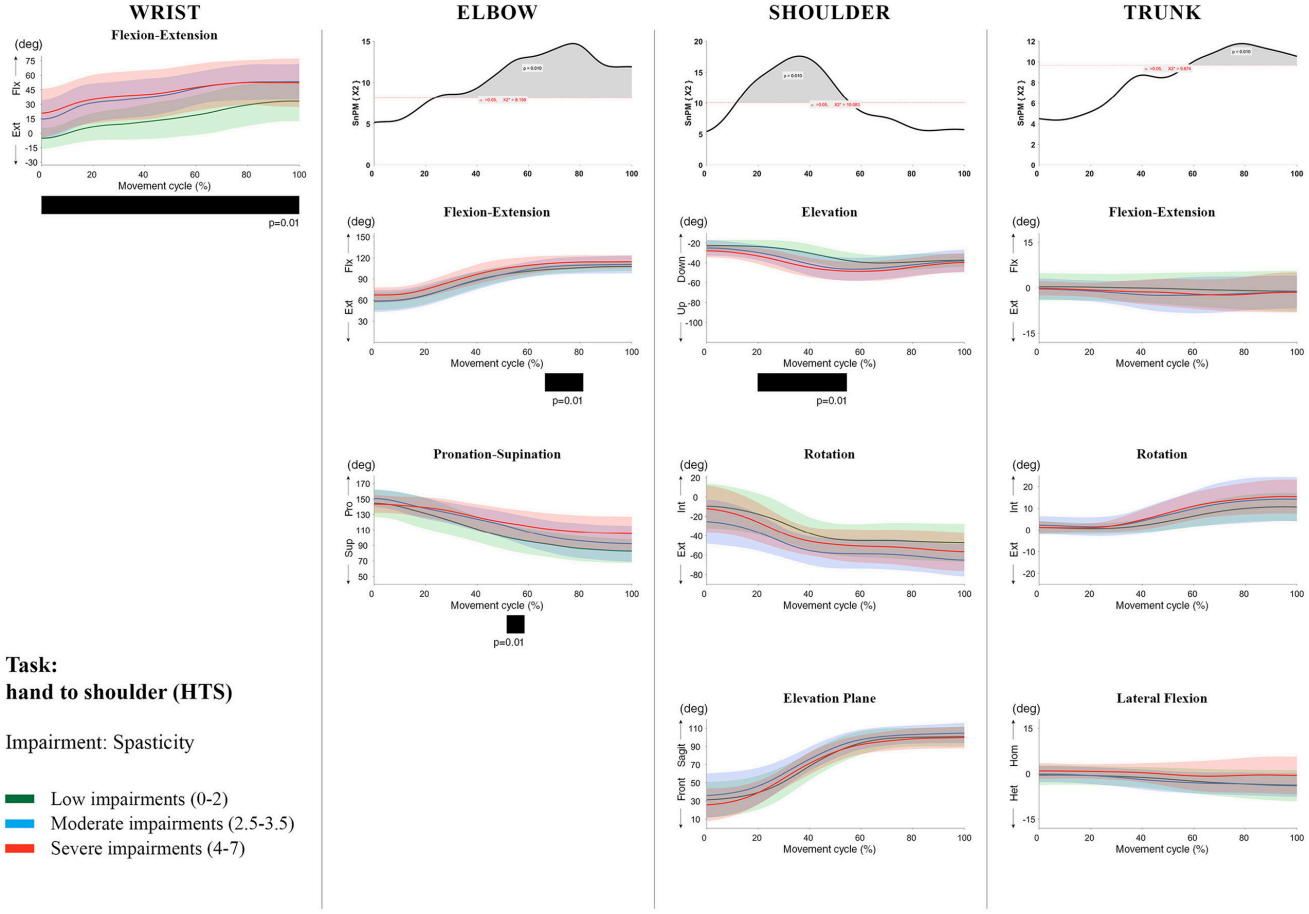
Impact of spasticity on UL movement patterns in children with uCP during the HTS task for all joint angles. Each column corresponds to a joint (from left to right: wrist, elbow, shoulder, and trunk). The top image of each column is the SPM output of the vector field analysis (non-parametric Canonical Correlation Analysis test, except for the wrist, where a non-parametric linear regression was computed). For visualization purposes, kinematic data was grouped according to the level of motor impairments, i.e., spasticity total score: low impairments (score between 0 and 2, i.e., values above percentile 75), moderate impairments (score between 2.5 and 3.5, i.e., values between percentile 25 and 75), and severe impairments (score between 4 and 7, i.e., values below percentile 25). Below, mean (bold line) and standard deviation (translucent area) of the low impairments (green), moderate impairments (blue), and severe impairments (red) of each vector component and the respective *post-hoc* SPM{t} output. The black bar under each kinematic profile indicates clusters of significant influence of the impairment level on the kinematic variable.

Results regarding the impact of muscle weakness on UL movement patterns can be found in Table 3 (all tasks), Figures 5–7 (RU, RGV, HTS), and Supplementary Material 2 (RF, RS, RGS, HTM, HTH). Similarly, results related to the impact of spasticity are presented in Table 4 (all tasks), Figures 8–10 (RU, RGV, HTS), and Supplementary Material 3 (RF, RS, RGS, HTM, HTH).

**Table 3:** Impact of muscle weakness on UL movement patterns in children with uCP.

			**RF**	**RU**	**RS**	**RGS**	**RGV**	**HTH**	**HTM**	**HTS**
WRIST	Flexion Extension	*n* clusters (*p*-value)	1 (0.01)	1 (0.01)	1 (0.01)	1 (0.01)	1 (0.01)	1 (0.01)	1 (0.01)	1 (0.01)
		% curve (range)	100% (0–100)	100% (0–100)	100% (0–100)	100% (0–100)	100% (0–100)	100% (0–100)	100% (0–100)	100% (0–100)
ELBOW	VECTOR FIELD	*n* clusters (*p*-value)	1 (0.01)	1 (0.01)	–	1 (0.01)	1 (0.01)	1 (0.01)	1 (0.01)	1 (0.01)
		% curve (range)	45% (55–100)	39% (61–100)		56% (44–100)	86% (14–100)	73% (27–100)	67% (33–100)	71% (29–100)
	Flexion Extension	*n* clusters (*p*-value)	1 (0.002)	1 (0.002)	–	1 (0.01)	1 (0.01)	–	–	–
		% curve (range)	40% (60–100)	38% (62–100)		49% (51–100)	56% (44–100)			
	Pro-supination	*n* clusters (*p*-value)	–	–	–	–	1 (0.01)	1 (0.01)	1 (0.01)	1 (0.01)
		% curve (range)					81% (19–100)	70% (30–100)	71% (29–100)	66% (34–100)
SHOULDER	VECTOR FIELD	*n* clusters (*p*-value)	1 (0.01)	1 (0.01)	1 (0.01)	1 (0.01)	1 (0.01)	1 (0.01)	1 (0.01)	1 (0.01)
		% curve (range)	100% (0–100)	100% (0–100)	74% (0–74)	100% (0–100)	100% (0–100)	41% (0–41)	100% (0–100)	97% (3–100)
	Elevation	*n* clusters (*p*-value)	1 (0.01)	1 (0.01)	1 (0.01)	1 (0.01)	1 (0.01)	1 (0.01)	1 (0.01)	1 (0.01)
		% curve (range)	56% (0–56)	38% (9–47)	60% (5–65)	35% (14–49)	35% (9–44)	39% (11–50)	72% (28–100)	88% (12–100)
	Rotation	*n* clusters (*p*-value)	–	2 (0.01; 0.01)	1 (0.01)	2 (0.01; 0.01)	–	1 (0.01)	–	–
		% curve (range)		28% 48% (0–28) (52–100)	100% (0–100)	18% 24% (14–32) (76–100)		8% (18–26)		
TRUNK	VECTOR FIELD	*n* clusters (*p*-value)	0	0	0	1 (0.01)	1 (0.02)	0	0	1 (0.01)
		% curve (range)				71% (29–100)	50% (50–100)			72% (28–100)
	Axial Rotation	*n* clusters (*p*-value)	–	–	–	1 (0.01)	1 (0.01)	–	–	1 (0.01)
		% curve (range)				85% (15–100)	51% (49–100)			42% (58–100)

*uCP, unilateral cerebral palsy; TDC, typically developing children; RF, reach forwards; RU, reach upwards; RS, reach sideways; RGS, reach-to-grasp a sphere; RGV, reach-to-grasp a vertical cylinder; HTH, hand-to-head; HTM, hand-to-mouth; HTS, hand-to-shoulder; n clusters, number of identified clusters; p-value, probability of the identified cluster: α < 0.05 vector field analysis, α < 0.017 (post post-hoc with three components), α < 0.025 (post post-hoc with two components); % curve, extent of the cluster over the movement cycle; range, start, and end point of the identified cluster*.

**Table 4:** Impact of spasticity on UL movement patterns in children with uCP.

			**RF**	**RU**	**RS**	**RGS**	**RGV**	**HTH**	**HTM**	**HTS**
WRIST	Flexion Extension	*n* clusters (*p*-value)	1 (0.01)	1 (0.01)	1 (0.01)	1 (0.002)	2 (0.01; 0.01)	1 (0.01)	1 (0.01)	1 (0.01)
		% curve (range)	100% (0–100)	100% (0–100)	100% (0–100)	100% (0–100)	62% 24% (0–62) (76–100)	16% (0–16)	100% (0–100)	100% (0–100)
ELBOW	VECTOR FIELD	*n* clusters (*p*-value)	1 (0.01)	1 (0.01)	–	1 (0.01)	1 (0.01)	1 (0.01)	1 (0.01)	1 (0.01)
		% curve (range)	44% (56–100)	42% (58–100)		49% (51–100)	73% (27–100)	71% (29–100)	61% (39–100)	76% (24–100)
	Flexion Extension	*n* clusters (*p*-value)	1 (0.01)	–	–	1 (0.01)	1 (0.01)	–	–	1 (0.01)
		% curve (range)	14% (86–100)			33% (68–100)	26% (74–100)			15% (66–81)
	Pro-supination	*n* clusters (*p*-value)	1 (0.01)	1 (0.01)	–	1 (0.01)	1 (0.01)	1 (0.01)	1 (0.01)	1 (0.01)
		% curve (range)	71% (29–100)	33% (58–91)		71% (29–100)	79% (21–100)	75% (25–100)	51% (49–100)	7% (51–58)
SHOULDER	VECTOR FIELD	*n* clusters (*p*-value)	1 (0.01)	1 (0.01)	1 (0.01)	1 (0.01)	2 (0.01; 0.01)	1 (0.02)	1 (0.01)	1 (0.01)
		% curve (range)	100% (0–100)	95% (5–100)	61% (0–61)	87% (13–100)	50% 40% (0–50) (60–100)	33% (7–40)	84% (16–100)	44% (12–56)
	Elevation	*n* clusters (*p*-value)	1 (0.01)	1 (0.01)	1 (0.01)	1 (0.01)	1 (0.01)	1 (0.01)	1 (0.01)	1 (0.01)
		% curve (range)	40% (10–50)	27% (18–45)	26% (24–50%)	37% (13–50)	27% (14–40)	21% (20–41)	72% (28–100)	35% (19–54)
	Elevation plane	*n* clusters (*p*-value)	1 (0.01)	1 (0.01)	–	1 (0.01)	1 (0.01)	–	–	–
		% curve (range)	42% (58–100)	48% (52–100)		60% (40–100)	34% (61–95)			
TRUNK	VECTOR FIELD	*n* clusters (*p*-value)	1 (0.01)	0	1 (0.01)	2 (0.04; 0.04)	1 (0.01)	0	0	1 (0.01)
		% curve (range)	35% (65–100)		54% (46–100)	12% 20% (0–12) (57–77)	63% (37–100)			42% (58–100)
	Axial Rotation	*n* clusters (*p*-value)	1 (0.01)	–	1 (0.01)	1 (0.01)	1 (0.01)	–	–	0
		% curve (range)	36% (64–100)		59% (41–100)	55% (45–100)	67% (33–100)			

*uCP, unilateral cerebral palsy; TDC, typically developing children; RF, reach forwards; RU, reach upwards; RS, reach sideways; RGS, reach-to-grasp a sphere; RGV, reach-to-grasp a vertical cylinder; HTH, hand-to-head; HTM, hand-to-mouth; HTS, hand-to-shoulder; n clusters, number of identified clusters; p-value, probability of the identified cluster: α < 0.05 vector field analysis, α < 0.017 (post hoc with three components), α < 0.025 (post post-hoc with two components); % curve, extent of the cluster over the movement cycle; range, start, and end point of the identified cluster*.

Increased muscle weakness at the level of the **wrist** significantly increased wrist flexion for all tasks (100%, *p* < 0.01). Increased spasticity also negatively impacted on wrist flexion (all tasks, 100%, *p* < 0.01), except for RGV (cluster 1: 0–62%, *p* < 0.01; cluster 2: 76–100%, *p* < 0.01), and HTH (0–16%, *p* < 0.01).

SPM vector field analysis at the **elbow** (two DoF) showed that both muscle strength and spasticity scores significantly influenced elbow kinematics in the second half of the movement cycle for all tasks (muscle weakness *p* = 0.01; spasticity *p* = 0.01), except for RS, where neither motor impairment influenced the movement patterns (*p* > 0.05). *Post-hoc scalar field analysis for muscle weakness* showed that this impairment mainly contributed to increased elbow flexion in the reaching and reach-to-grasp tasks (44–100%, *p* = 0.01), whereas its contribution to increased pronation could be clearly observed in the gross motor tasks during a large extent of the movement cycle (~30–100%, *p* = 0.01). RGV was the only task in which muscle weakness negatively influenced both elbow supination (19–100%, *p* = 0.01) and extension (44–100%, *p* = 0.01). *Post-hoc scalar field analysis for spasticity* showed that this factor mainly contributed to increased pronation in all tasks toward the end of the movement cycle (58–100%, *p* = 0.01), except in RS. The influence of spasticity on increased elbow flexion was visible in the last third of the movement cycle during reaching and reach-to-grasp (RF, RGS, and RGV: ~75–100%, *p* < 0.01).

At the level of the **shoulder** (three DoF), both motor impairments had a negative impact on shoulder kinematics, ranging from at least 0–40% to up to 100% of the movement cycle in all tasks (muscle strength *p* = 0.01; spasticity *p* < 0.02). *Post-hoc scalar field analysis for muscle strength* showed that shoulder rotation and shoulder elevation were primarily responsible for the vector field results. Location of impact of muscle weakness on shoulder rotation varied among the tasks. Muscle weakness resulted in increased external rotation during RGS (cluster 1: 14–32%, cluster 2:76–100%, *p* = 0.01), RU (cluster 1: 0–28%, cluster 2: 52–100%, *p* = 0.01), RS (100%, *p* = 0.01), and HTH (18–26%, *p* = 0.01). Muscle weakness also explained the decreased shoulder elevation at the beginning of the movement cycle during reaching and HTH (0–50%, *p* = 0.01), and during HTM and HTS (28–100%, *p* = 0.01). The elevation plane was not significantly affected by muscle strength in any of the tasks. *Post-hoc scalar field analysis for spasticity* showed that shoulder elevation and elevation plane were mainly responsible for the significant vector field results. Spasticity resulted in less shoulder elevation in the middle part of the movement cycle (all tasks, approx. between 20 and 50%, *p* = 0.01) and increased elevation in the frontal plane second half of the movement cycle during reaching and reach-to-grasp (approx. between 52 and 100%, *p* = 0.01). Shoulder rotation was not significantly influenced by spasticity.

SPM vector field analysis for the **scapula** (three DoF) showed no significant influence of muscle weakness or spasticity on scapular kinematics.

For the **trunk** (three DoF), muscle weakness had a negative influence on trunk kinematics during reach-to-grasp and HTS toward the end of the movement cycle (approx. between 50 and 100%, *p* < 0.02). Increased spasticity also affected trunk movement patterns during RF, RS, both reach-to-grasp tasks and HTS (approx. between 57 and 100%, *p* < 0.04). *Post-hoc scalar field analysis* showed that axial rotation was the only responsible for the vector field results for both motor impairments. Muscle weakness resulted in increased trunk inward rotation toward the end of the movement cycle (reach-to-grasp tasks, HTS; approx. between 58 and 100%, *p* = 0.01). Increased trunk inward rotation toward the end of the movement was also seen in case of increased spasticity (RF, reach-to-grasp tasks; approx. between 64 and 100%, *p* = 0.01). In contrast, during RS, children with increased spasticity scores showed increased trunk outward rotation (41–100%, *p* = 0.01).

## Discussion

In this study, we used a statistical approach, i.e., vector field analysis based on SPM1D, (1) to examine differences in movement patterns between a large cohort of children with uCP and TDC during the execution of different UL tasks; and (2) to explore the relation between distal motor impairments and UL movement patterns in children with uCP.

The SPM vector field analysis identified pronounced differences between children with uCP and TDC, including increased wrist flexion, elbow flexion and pronation. These differences were present for most tasks and during a large extent of the movement cycle. Results are in line with previous studies reporting deviant distal kinematics in children with uCP, i.e., increased wrist or elbow flexion at the start or end of the movement, reduced elbow supination at the end of the movement and reduced elbow total range of motion (Kreulen et al., 2007; Jaspers et al., 2011a,c; Riad et al., 2011; Butler and Rose, 2012; Klotz et al., 2014). Proximally, children with uCP also showed deviant movement patterns compared to TDC during all UL tasks, mostly consisting of (1) increased shoulder external rotation, decreased shoulder elevation and a preference for movements in the frontal plane, (2) increased scapular anterior tilting, medial rotation and protraction, and (3) increased inwards trunk axial rotation. Interestingly, children with uCP showed most scapular deficits at rest and during movement initiation, whereas the second part of the movement was mostly characterized by deviant shoulder kinematics. For example, during HTS, children with uCP initiated the movement with large scapular deficits, which coincided with increased shoulder elevation in the frontal plane. Children only switched to the sagittal plane when approaching the contralateral shoulder (second half of the movement cycle), which was combined with increased external shoulder rotation. Such movement deviations can occur as a compensation for the lack of elbow supination. Previous literature, based on either extracted scalar metrics (end point angles, total range of motion) or summary indices, failed to identify differences in shoulder kinematics between uCP and TDC for most tasks (MacKey et al., 2006; Jaspers et al., 2011c). Lastly, movement deviations at the trunk consisted of increased inward rotation during all the tasks, except RS, where children with uCP showed an increased outward rotation. The increased trunk rotations could also be considered a compensation for the distal deficits, which is in agreement with previous studies (Kreulen et al., 2007; Jaspers et al., 2011a). In general, it appears that for wrist, elbow and trunk kinematics, scalar metrics and summary indices might be sufficient to capture differences between children with uCP and TDC, although SPM1D was able to more specifically map the extent and the location of these differences over the movement cycle. Moreover, SPM1D analysis shows higher sensitivity to detect differences in kinematics at the shoulder and scapula compared to scalar metrics.

Current study results also showed that increased spasticity and muscle weakness explained the deviant wrist and elbow kinematics in the majority of tasks. Muscle weakness negatively influenced active elbow extension in the reaching and reach-to-grasp tasks, as well as active supination during the gross motor tasks. Spasticity also negatively influenced the supination deficit during reaching. The results at the level of the wrist and elbow are in agreement with previous literature (Jaspers et al., 2011c; Mailleux et al., 2017), which showed low to strong correlations between motor impairments and UL kinematics (either extracted parameters or summary indices). Remarkably, both muscle weakness and spasticity explained deviant shoulder kinematics, i.e., muscle weakness affected external rotation and elevation kinematics, whereas spasticity mostly influenced arm elevation and elevation plane kinematics. Also, both motor impairments were related to increased trunk deviations, with a stronger influence of spasticity on trunk rotation. This has recently been reported by Mailleux et al. who found low to moderate correlations with some extracted trunk kinematic parameters (Mailleux et al., 2017). The negative impact of distal motor impairments on proximal shoulder and trunk kinematics strongly supports the idea that these proximal movement patterns are compensations of the distal motor impairments. The lack of significant results at the level of the scapula in the present study suggests that scapula kinematics might be influenced by more proximal motor impairments. Efficient shoulder and scapula movements require an adequate stability and coordination of the scapulathoracic and glenohumeral joint and the surrounding muscle complex (Paine and Voight, 2013). The reported scapular deficits at rest could be caused by altered muscle length and muscle activation patterns of the scapulathoracic and glenohumeral muscles (McClure et al., 2001; Ludewig and Reynolds, 2009), as seen in stroke survivors (De Baets et al., 2016). Thus far, only the study of Mailleux et al. reported the relation between muscle weakness and kinematic deficits at each joint angle (Mailleux et al., 2017). The authors assessed scapula kinematics in three tasks and found no relation between UL muscle weakness and discrete parameters, except for a moderate correlation with the active range of motion of scapula rotation in one task. Their results implied that weaker children performed the task hand-to-mouth with increased lateral rotation, which is in contrast with our results. However, extracting specific scalars from a time-varying trajectory, i.e., kinematic waveform, has been suggested to increase the probability of false positive rate (Pataky et al., 2016b). The strength of SPM lies in decreasing the chances of incorrectly rejecting the null hypothesis by adjusting the threshold to the real number of comparisons. Therefore, we hypothesize that the moderate correlation found in the study of Mailleux et al may be due to the so called “regional focus bias” (Pataky et al., 2013). Nevertheless, firm conclusions based on our results cannot be drawn given the lack of an evaluation of proximal motor impairments. In summary, both studies by Jaspers et al and Mailleux et al previously reported a correlation between UL movement pathology and muscle weakness, although only Mailleux et al also found a correlation with spasticity (Jaspers et al., 2011c; Mailleux et al., 2017). However, both studies report deviations in UL movement patterns by extracting scalars or by computing summary indices of movement pathology (Jaspers et al., 2011c).

Overall, our results specifically highlight the importance of taking the entire movement cycle at the individual joint level into account to avoid an underestimation of the influence of underlying motor impairments on UL movement deviations. Interestingly, during the reaching tasks, muscle weakness mainly affected elbow extension, whereas during the gross motor tasks, muscle weakness mostly affected supination. We hypothesize that this is due to the muscle recruitment that each task requires. As we expected, for the RGV task, both muscle weakness and spasticity explained the elbow extension and supination deficits, given that this task simultaneously requires both motion components of elbow extension and supination, which are challenging for children with uCP. These results highlighted a task dependent influence of muscle weakness and spasticity and the relevance of choosing the right tasks for this population.

Current study results might have some interesting implications with respect to UL therapy planning in children with uCP. First, the fact that both spasticity and muscle weakness of the elbow and wrist have a negative impact on UL movement patterns, supports the use of interventions specifically targeting these impairments, such as Botulinum Neurotoxin-A (Park and Rha, 2006; Kreulen et al., 2007; Fitoussi et al., 2011) or functional strength training (Rameckers et al., 2008). SPM1D analysis would allow capturing the impact of these interventions at different levels of the UL kinematic chain. This might further aid in fine-tuning targeted interventions for the individual child with uCP. Second, the predominant distal impairments that are typically seen in children with uCP have thus far dominated UL rehabilitation programs such as Constraint-Induced Movement Therapy (Hoare et al., 2007; Eliasson et al., 2014), bimanual interventions (Gordon et al., 2007), or a combination of both (Gordon, 2011). Whilst it has been shown that treatment at the distal level may improve proximal movement patterns (shoulder and trunk; Kreulen et al., 2007; Fitoussi et al., 2011), our results suggest that these children might also benefit from scapulathoracic and glenohumeral stabilization training. These new insights in the relationship between motor impairments and movement patterns may provide a rationale for specific interventions targeting these motor impairments. However, further studies combining this information with clinical assessment scales are required to investigate the benefits of an integrated approach with respect to targeted treatment planning.

Finally, whilst we did not directly compare UL movement patterns between the different tasks, we do believe that current study results allow formulating guidelines regarding task selection in children with uCP. First of all, a movement protocol should challenge UL motor performance in a variety of ways, depending on the individual child's functional potential. This requires the inclusion of a non-grasping task for those children with limited or no grasping capabilities. Our results showed most pronounced kinematic differences between children with uCP and TDC during the reaching upwards (RU) task, where UL movement deficits were strongly influenced by both spasticity and muscle weakness. Among the reach-to-grasp tasks, grasping a vertical cylinder (RGV) elicited most differences between children with uCP and TDC, and kinematics were also strongly negatively affected by both motor impairments. For the gross motor tasks, our results point toward the use of HTS, as this task additionally identified most differences at the level of the scapula. This set of tasks (RU, RGV, HTS) will ensure a complete evaluation of the UL and will provide sufficient and comprehensive information about the impact of motor impairments on UL movement patterns in children with uCP. Furthermore, our analysis identified specific deviant parts of the movement pattern, i.e., clusters, which may help establishing the basis for further studies. Combining these identified clusters (regions of interest, Pataky et al., 2016a), possibly together with spatiotemporal parameters and extracted scalars will permit further hypothesis driven research. Moreover, the implementation of dimensionality reduction tool [Principal Component Analysis (PCA), Independent Component Analysis (ICA), or kernel Principal Component Analysis (kPCA)] and/or machine learning tools [Artificial Neural Network (ANN), Support Vector Machines (SVM), or Self-Organizing Maps (SOM)] would be of interest to classify movement patterns. The potential merit of these approaches has already been demonstrated in the field of clinical biomechanics (Ferber et al., 2016) as well as to assess treatment response predictions after lower limb surgical interventions in children with CP (Reinbolt et al., 2009). Such progress is crucial to improve the interpretation of the vast amount of data this assessment offers and will thereby undoubtedly further contribute to the clinical implementation of UL-3DMA.

This study also warrants some critical reflections. First, spasticity and muscle weakness were measured with ordinal scales. Although the MAS is the most commonly used scale to measure spasticity in clinical practice and its reliability has been established (Bohannon and Smith, 1987; Klingels et al., 2010), some controversy regarding the value and accuracy of the MAS does exist (Pandyan et al., 2003; Fleuren et al., 2010). An instrumented spasticity assessment, similar to the one available for the lower limbs (Bar-On et al., 2014), would be a valuable addition in UL research. Secondly, spasticity and muscle weakness were only assessed distally (elbow and wrist). However, proximal motor deficits (at the level of the shoulder, scapula, or trunk) may also have a negative contribution to UL movement patterns. Given that the current study did not include a proximal evaluation of these motor deficits, we cannot fully discriminate the contribution of distal vs. proximal deficits to deviant UL movement patterns. It would be therefore valuable to investigate whether proximal impairments also play a role in proximal and distal movement patterns. This would allow the identification of other factors that could complement current treatment approaches. Third, spasticity and muscle weakness were measured in a static position, which may not reflect the dynamic factor of muscle (dys)function during motion (Crenna, 1998; van der Krogt et al., 2010). Including electromyography measures will contribute to a better understanding of the mechanisms of dynamic spasticity on UL movement patterns in children with uCP. Lastly, although we performed vector field analysis and took into account the covariance among the vector components, our *post-hoc* comparisons were computed with a *t*-test and linear regression. These latter tests do not account for vector component covariances and should thus be interpreted with caution. However, the development of SPM1D is still ongoing and more suitable *post-hoc* analysis will be offered in the near future.

To the best of our knowledge, this is the first study that used vector field analysis over the continuum of the movement cycle to investigate UL movement patterns in a large cohort of children with uCP and TDC. We found that children with uCP presented with deviant UL movement patterns compared to TDC at the level of the wrist, elbow, shoulder, scapula, and trunk. In general, results of the current study show the importance of investigating the entire movement cycle to better understand where the deficits are most pronounced during different UL movements. Moreover, UL kinematic deviations were also strongly negatively influenced by distal muscle weakness and spasticity for all joints, except for the scapula, where other factors such as scapulathoracic muscle activation might play a role. Finally, based on current study results, we would recommend three UL tasks, i.e., reaching upwards, reach-to-grasp a vertical cylinder and hand-to-shoulder, as a comprehensive assessment protocol that allows mapping deviant UL movement patterns in children with uCP. Such protocol reduction will facilitate the implementation of UL-3DMA in a clinical setting, which will lead to a better understanding of UL movement pathology and ensure a more detailed and individualized treatment planning in children with uCP, to ultimately warrant that the child reaches its full functional potential.

## Author contributions

This study was designed by CS, KD, KK, EJ, and HF. CS, LM, and EJ were responsible for all data collection and processing. CS performed all data analysis with SPM1D. All authors contributed to the interpretation of the results and gave their critical views regarding the revision and editing of the manuscript, which was written by CS. All authors approved the final version of the manuscript and agreed to be accountable for the content of the study.

### Conflict of interest statement

The authors declare that the research was conducted in the absence of any commercial or financial relationships that could be construed as a potential conflict of interest.
