# Supplementary material for: Negative Influence of Motor Impairments on Upper Limb Movement Patterns in Children with Unilateral Cerebral Palsy. A Statistical Parametric Mapping Study
Source: Front Hum Neurosci. 2017 Oct 5;11:482. doi: 10.3389/fnhum.2017.00482 (PMC5633911; doi:10.3389/fnhum.2017.00482)
Supplement: Supplementary file 1 [file Image1.PDF]

Supplementary Material 1

Negative influence of motor impairments on upper limb movement patterns in children with unilateral cerebral palsy. A statistical parametric mapping study

Simon-Martinez, C*; Jaspers, E; Mailleux, L; Desloovere, K; Vanrenterghem, J; Ortibus, E; Molenaers, G; Feys, H; Klingels, K

*** Correspondence:** Cristina Simon-Martinez; cristina.simon@kuleuven.be

Comparison between children with uCP and TDC during the RF, RS, RGS, HTH and HTM tasks for all joint angles. Each column corresponds to a joint (from left to right: wrist, elbow, shoulder, scapula and trunk). The top image of each column is the SPM output of the vector field analysis (Hoteling's test, except for the wrist, where a t-test was computed). Below, mean (bold line) and standard deviation (translucent area) of the TDC (green) and uCP (blue) of each vector component, and the respective post-hoc SPM{t} output.

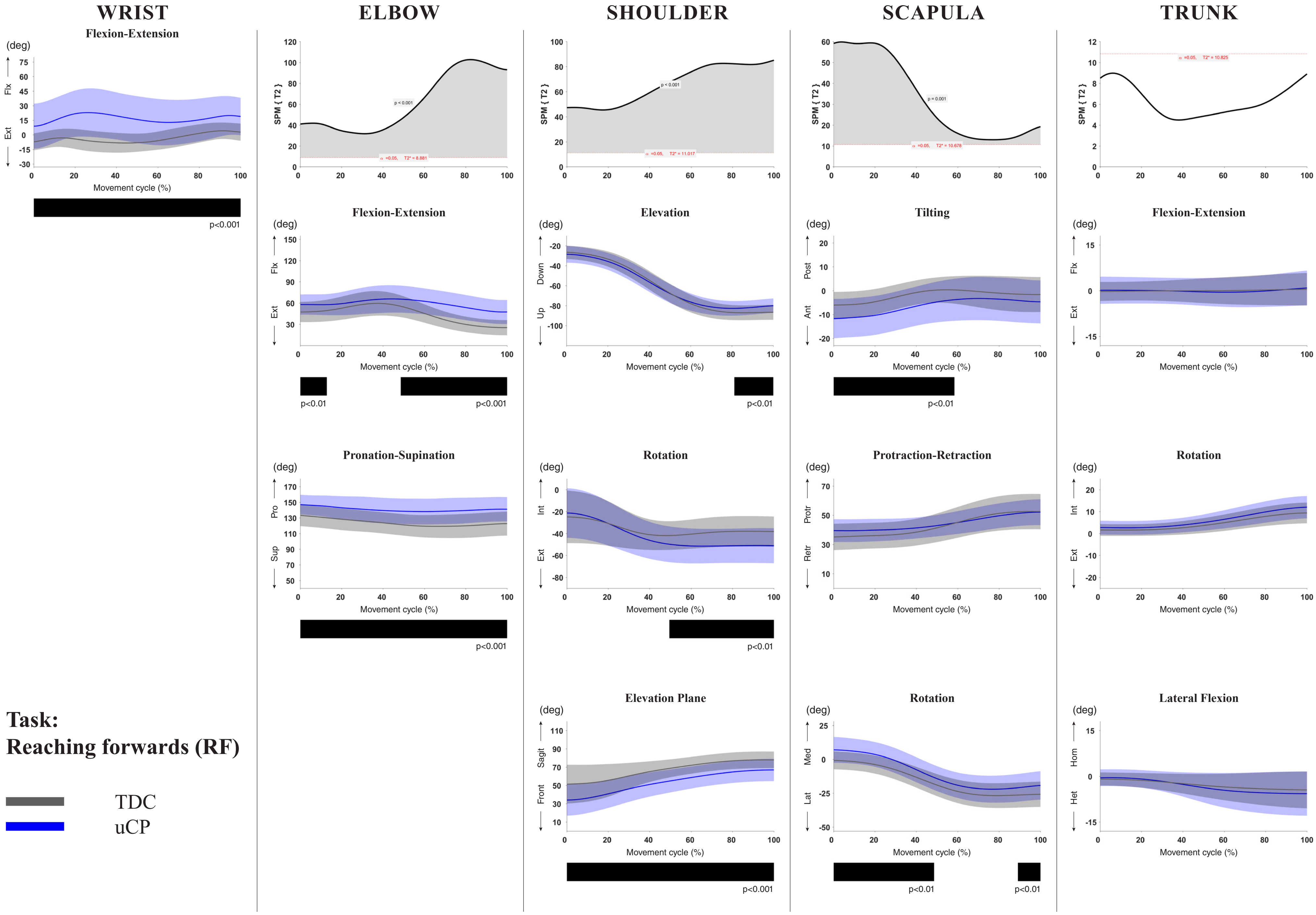
WRIST
ELBOW
SHOULDER
SCAPULA
TRUNK
Flexion-Extension
(deg)
Flx
Ext
Movement cycle (%)
p<0.001
SPM {T2}
p < 0.001
p = 0.001
Flexion-Extension
p<0.01
p<0.001
Elevation
Down
Up
p<0.01
Tilting
Post
Ant
p<0.01
Flexion-Extension
Pronation-Supination
Pro
Sup
p<0.001
Rotation
Int
Ext
p<0.01
Protraction-Retraction
Protr
Retr
Rotation
Int
Ext
Elevation Plane
Sagit
Front
p<0.001
Rotation
Med
Lat
p<0.01
p<0.01
Lateral Flexion
Hom
Het
Task:
Reaching forwards (RF)
TDC
uCP

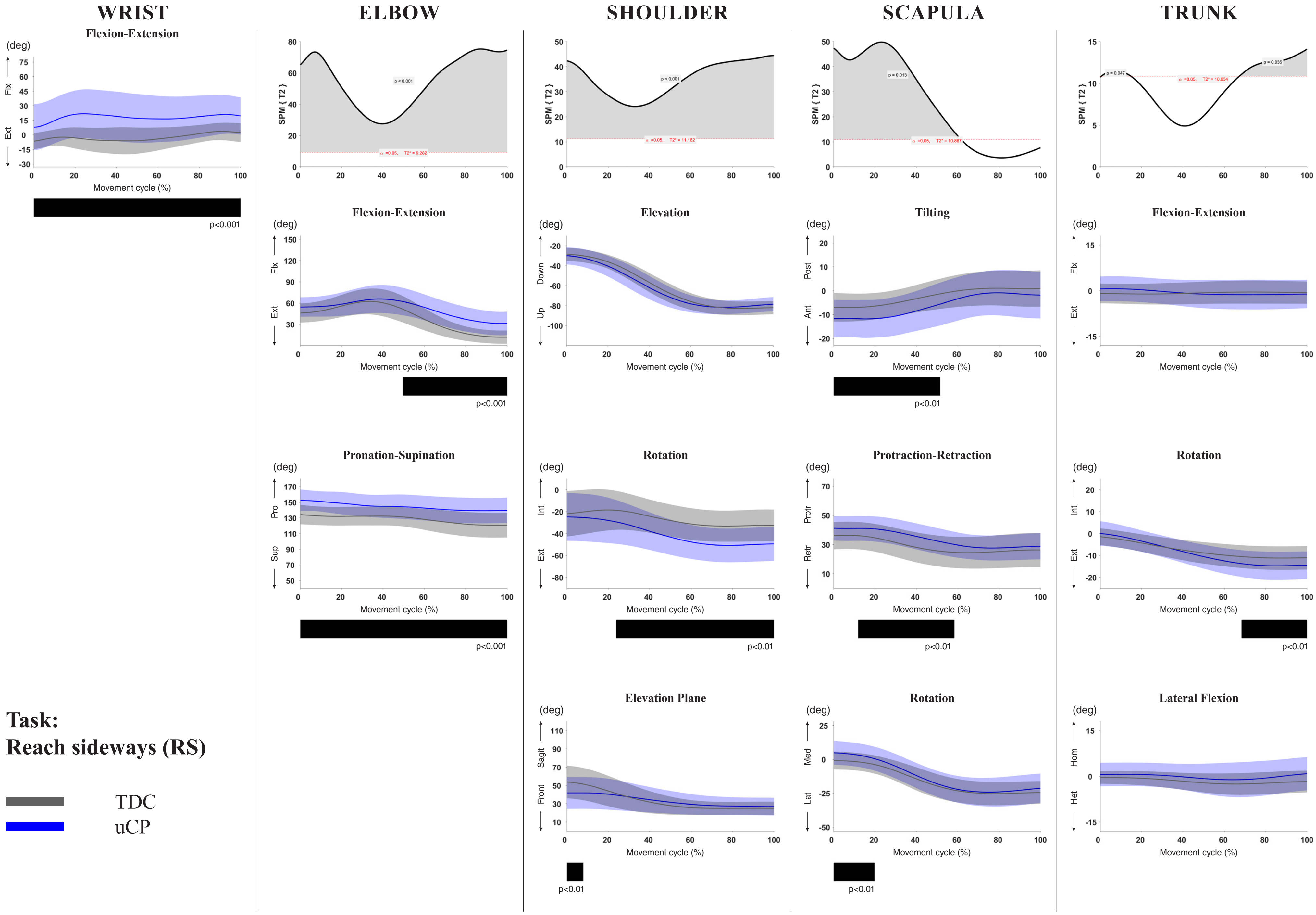
WRIST
ELBOW
SHOULDER
SCAPULA
TRUNK
Flexion-Extension
(deg)
Flx
Ext
Movement cycle (%)
p<0.001
SPM { T2 }
p < 0.001
α =0.05, T2* = 9.282
α =0.05, T2* = 11.182
p = 0.013
α =0.05, T2* = 10.867
p = 0.047
p = 0.035
α =0.05, T2* = 10.854
Elevation
Down
Up
Tilting
Post
Ant
p<0.01
Pronation-Supination
Pro
Sup
Rotation
Int
Ext
Protraction-Retraction
Protr
Retr
Elevation Plane
Sagit
Front
Med
Lat
Lateral Flexion
Hom
Het
Task:
Reach sideways (RS)
TDC
uCP

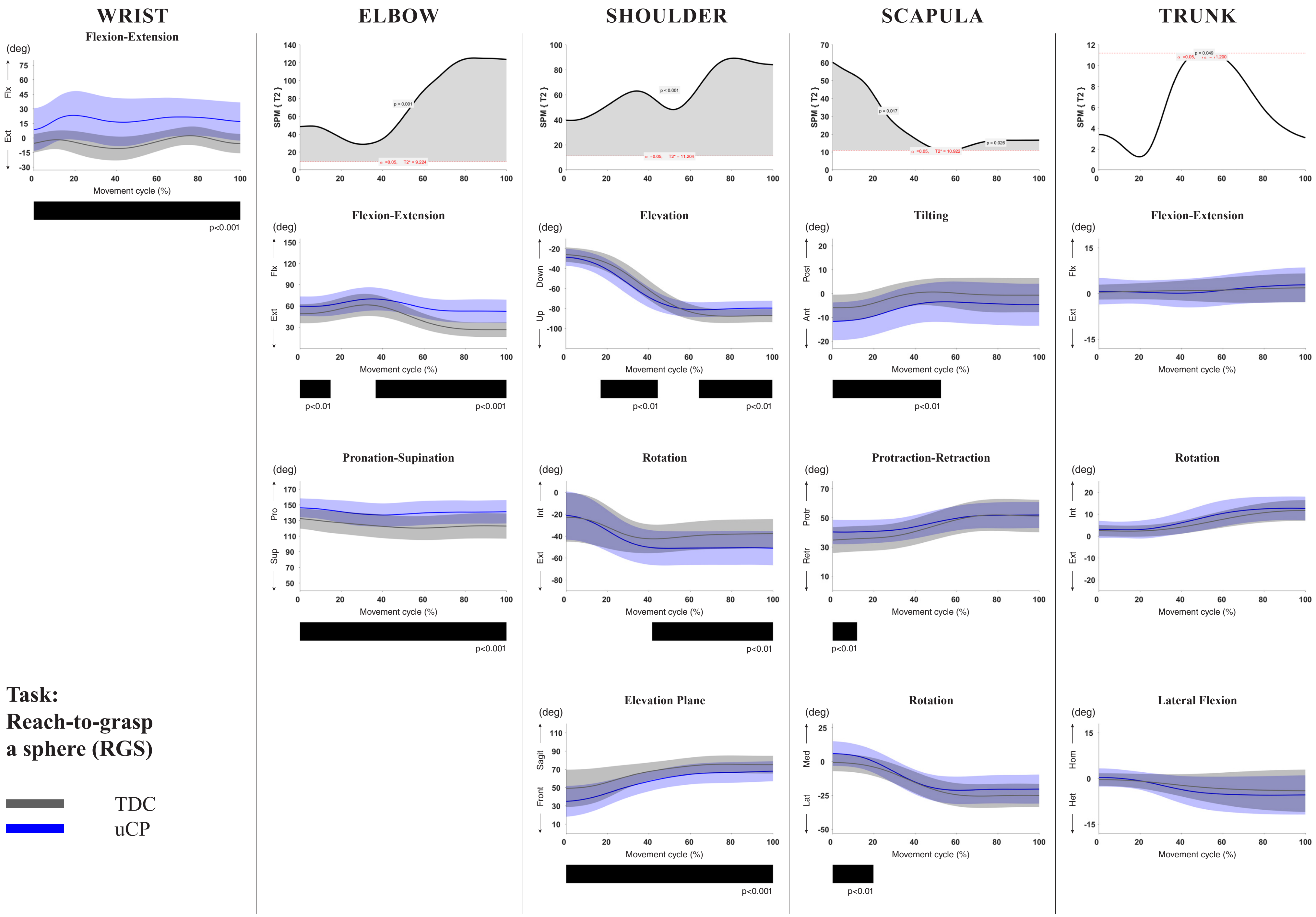
WRIST
ELBOW
SHOULDER
SCAPULA
TRUNK
Flexion-Extension
(deg)
Flx
Ext
Movement cycle (%)
p<0.001
SPM {T2}
Flexion-Extension
p<0.01
p<0.001
Elevation
Down
Up
p<0.01
p<0.01
Tilting
Post
Ant
p<0.01
Flexion-Extension
Pronation-Supination
Pro
Sup
p<0.001
Rotation
Int
Ext
p<0.01
Protraction-Retraction
Protr
Retr
p<0.01
Rotation
Elevation Plane
Sagit
Front
p<0.001
Rotation
Med
Lat
p<0.01
Lateral Flexion
Hom
Het
Task:
Reach-to-grasp
a sphere (RGS)
TDC
uCP

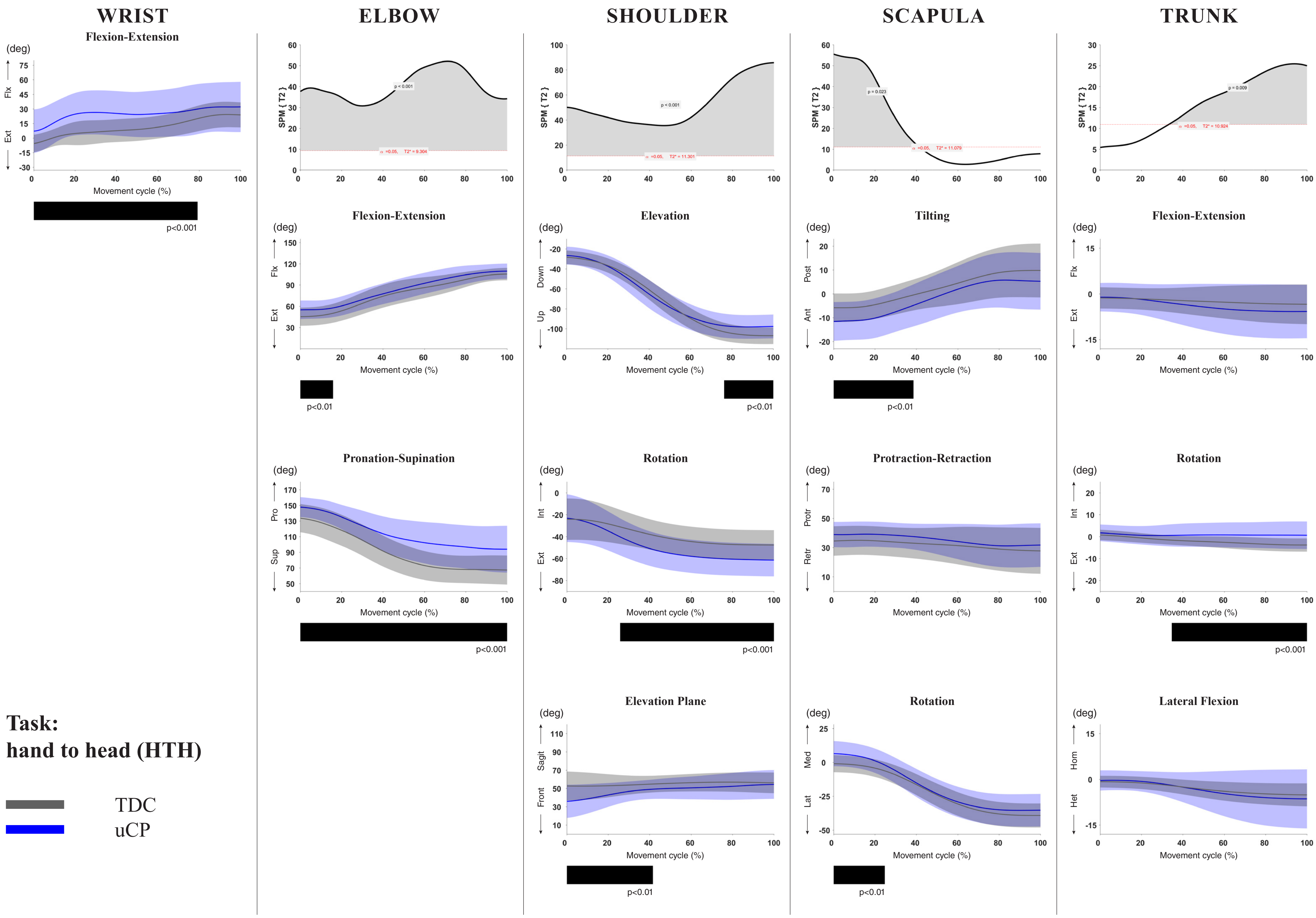
WRIST
ELBOW
SHOULDER
SCAPULA
TRUNK
Flexion-Extension
(deg)
Flx
Ext
Movement cycle (%)
p<0.001
SPM{T2}
p < 0.001
α =0.05, T2* = 9.304
p < 0.001
α =0.05, T2* = 11.301
p = 0.023
α =0.05, T2* = 11.079
p = 0.009
α =0.05, T2* = 10.924
Flexion-Extension
p<0.01
Elevation
Down
Up
p<0.01
Tilting
Post
Ant
p<0.01
Flexion-Extension
Pronation-Supination
Pro
Sup
p<0.001
Rotation
Int
Ext
p<0.001
Protraction-Retraction
Protr
Retr
Rotation
p<0.001
Elevation Plane
Sagit
Front
p<0.01
Rotation
Med
Lat
p<0.01
Lateral Flexion
Hom
Het
Task:
hand to head (HTH)
TDC
uCP

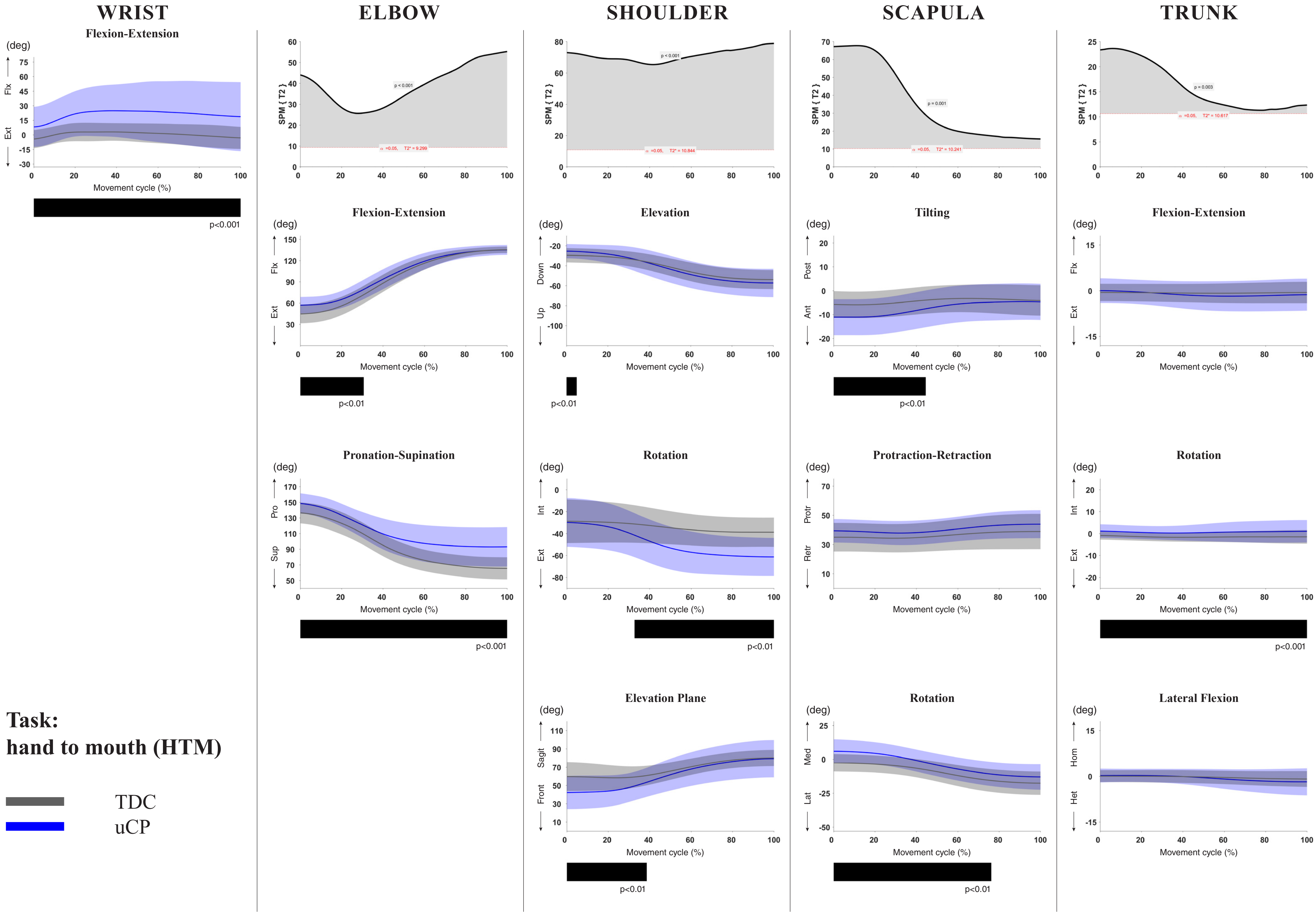
WRIST
ELBOW
SHOULDER
SCAPULA
TRUNK
Flexion-Extension
(deg)
Flx
Ext
Movement cycle (%)
p<0.001
SPM { T2 }
Flexion-Extension
p<0.01
Pronation-Supination
Pro
Sup
p<0.001
Elevation
Down
Up
p<0.01
Rotation
Int
Ext
p<0.01
Elevation Plane
Sagit
Front
p<0.01
Tilting
Post
Ant
p<0.01
Protraction-Retraction
Protr
Retr
Rotation
Med
Lat
p<0.01
Flexion-Extension
Rotation
p<0.001
Lateral Flexion
Hom
Het
Task:
hand to mouth (HTM)
TDC
uCP
