# Supplementary material for: Negative Influence of Motor Impairments on Upper Limb Movement Patterns in Children with Unilateral Cerebral Palsy. A Statistical Parametric Mapping Study
Source: Front Hum Neurosci. 2017 Oct 5;11:482. doi: 10.3389/fnhum.2017.00482 (PMC5633911; doi:10.3389/fnhum.2017.00482)
Supplement: Supplementary file 2 [file Image2.pdf]

Supplementary Material 2

Negative influence of motor impairments on upper limb movement patterns in children with unilateral cerebral palsy. A statistical parametric mapping study

Simon-Martinez, C*; Jaspers, E; Mailleux, L; Desloovere, K; Vanrenterghem, J; Ortibus, E; Molenaers, G; Feys, H; Klingels, K

*** Correspondence:** Cristina Simon-Martinez; cristina.simon@kuleuven.be

Impact of muscle weakness on UL movement patterns in children with uCP during the RF, RS, RGS, HTH and HTM tasks for all joint angles. Each column corresponds to a joint (from left to right: wrist, elbow, shoulder and trunk). The top image of each column is the SPM output of the vector field analysis (non-parametric Canonical Correlation Analysis test, except for the wrist, where a non-parametric linear regression was computed). For visualization purposes, kinematic data was grouped according to the level of motor impairments, i.e. muscle weakness total score: low impairments (score between 12 and 15, i.e. values above percentile 75), moderate impairments (score between 10.5 and 11.5, i.e. values between percentile 25 and 75), and severe impairments (score between 5.5 and 10, i.e. values below percentile 25). Below, mean (bold line) and standard deviation (translucent area) of the low impairments (green), moderate impairments (blue) and severe impairments (red) of each vector component and the respective post-hoc SPM{t} output. The black bar under each kinematic profile indicates clusters of significant influence of the impairment level on the kinematic variable.

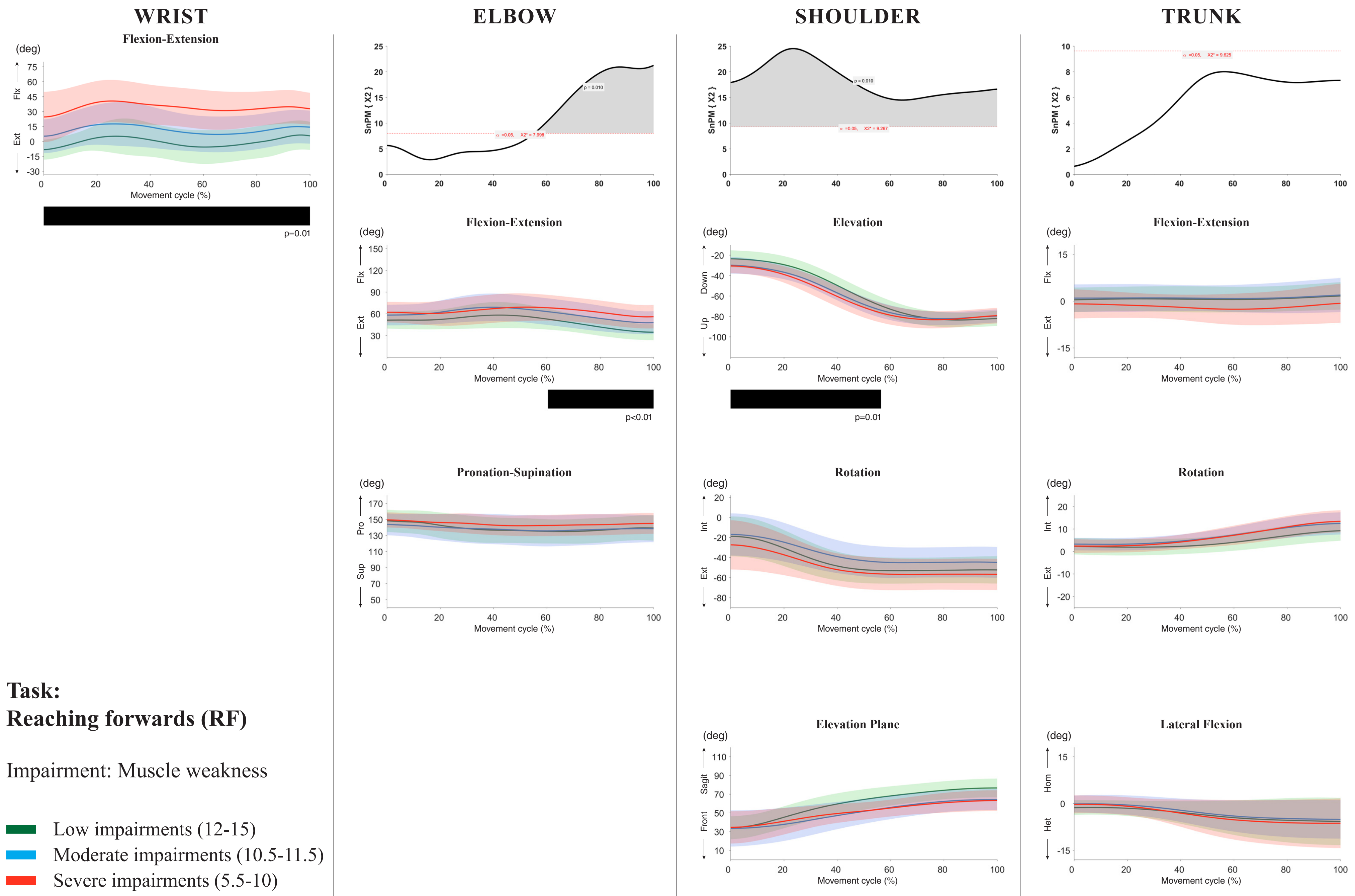

WRIST
ELBOW
SHOULDER
TRUNK
Flexion-Extension
(deg)
Flx
Ext
Movement cycle (%)
p=0.01
SnPM { X2 }
p = 0.010
Flexion-Extension
p<0.01
Elevation
Down
Up
p=0.01
Flexion-Extension
Pronation-Supination
Pro
Sup
Rotation
Int
Ext
Rotation
Elevation Plane
Sagit
Front
Lateral Flexion
Hom
Het
Task:
Reaching forwards (RF)
Impairment: Muscle weakness
Low impairments (12-15)
Moderate impairments (10.5-11.5)
Severe impairments (5.5-10)

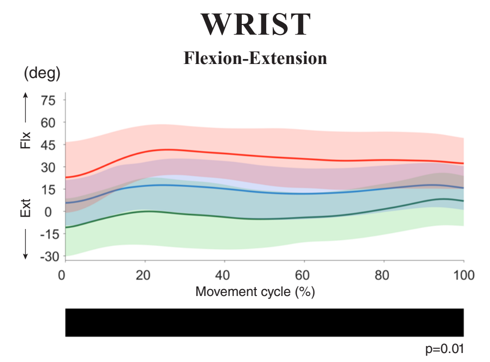

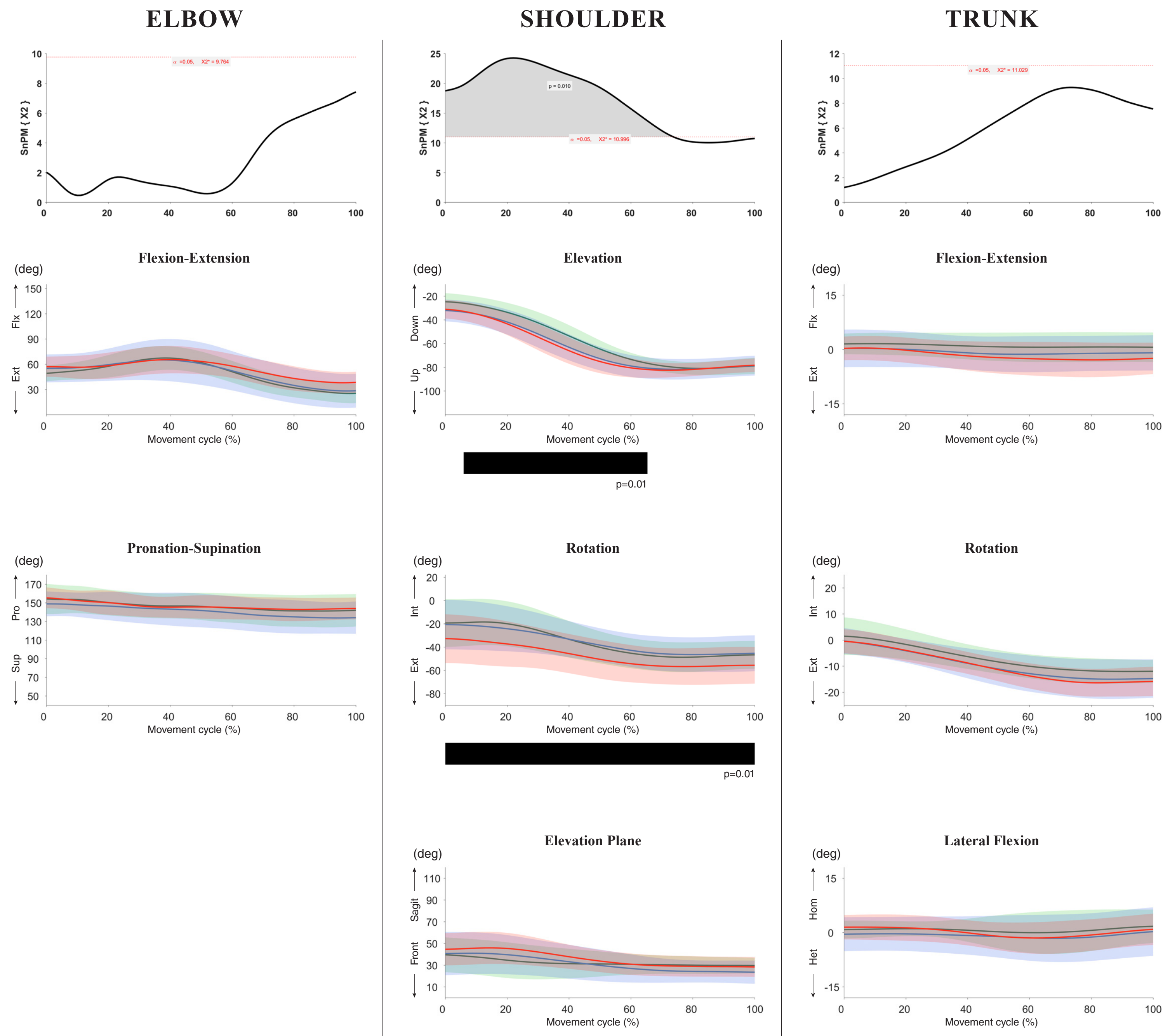

Task:
Reach sideways (RS)

Impairment: Muscle weakness

- Low impairments (12-15)
- Moderate impairments (10.5-11.5)
- Severe impairments (5.5-10)

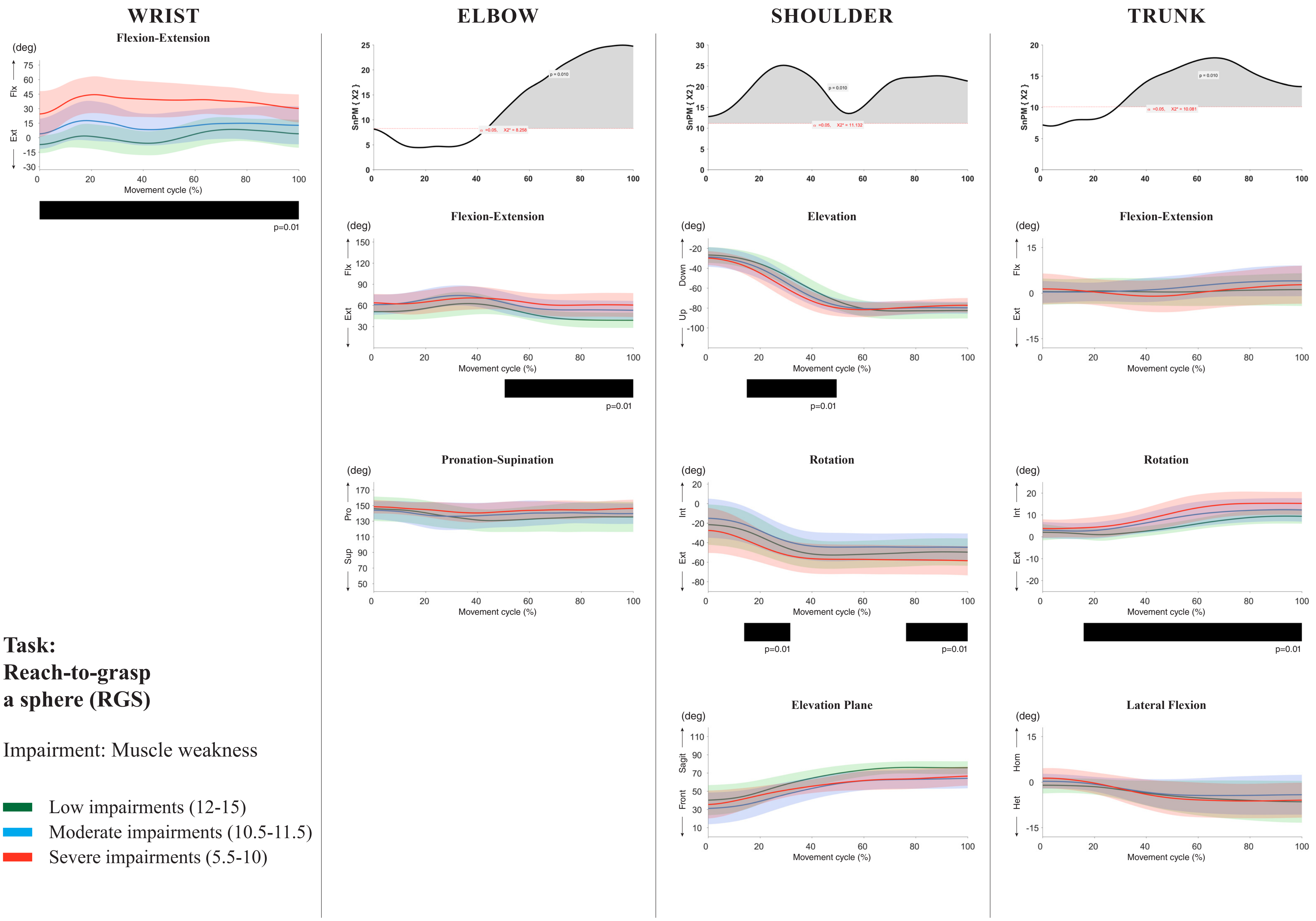

Task:
Reach-to-grasp
a sphere (RGS)

Impairment: Muscle weakness

- Low impairments (12-15)
- Moderate impairments (10.5-11.5)
- Severe impairments (5.5-10)

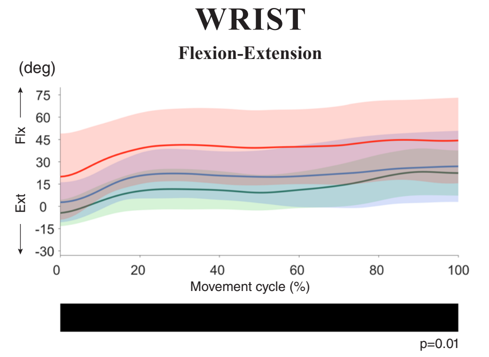

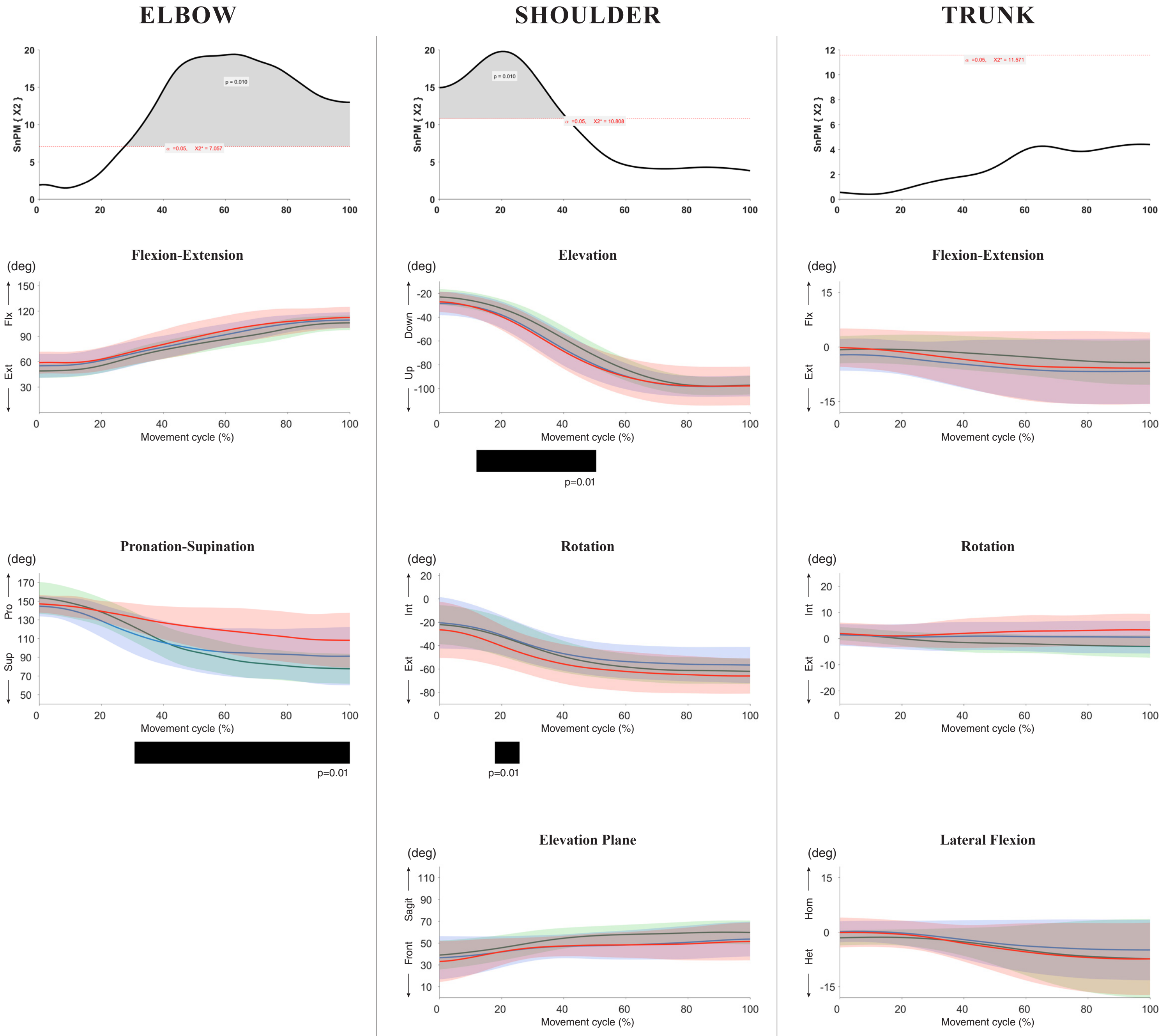

Task:
hand to head (HTH)

Impairment: Muscle weakness

- Low impairments (12-15)
- Moderate impairments (10.5-11.5)
- Severe impairments (5.5-10)

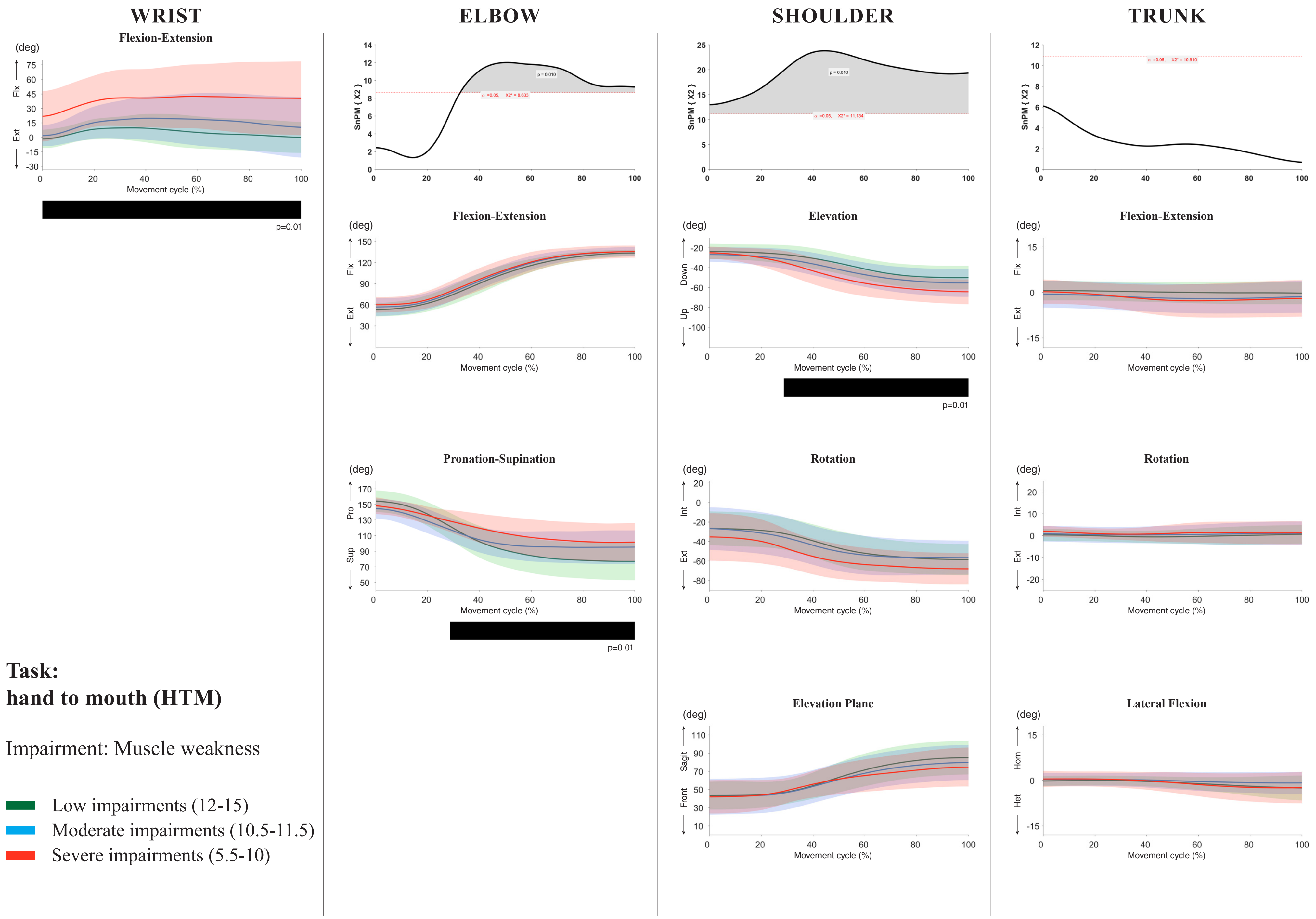

WRIST
ELBOW
SHOULDER
TRUNK
Flexion-Extension
(deg)
Flx
Ext
Movement cycle (%)
p=0.01
SnPM { X2 }
p = 0.010
Flexion-Extension
Elevation
Down
Up
Pronation-Supination
Pro
Sup
Rotation
Int
Ext
Elevation Plane
Sagit
Front
Lateral Flexion
Hom
Het
Task:
hand to mouth (HTM)
Impairment: Muscle weakness
Low impairments (12-15)
Moderate impairments (10.5-11.5)
Severe impairments (5.5-10)
