# Supplementary material for: Negative Influence of Motor Impairments on Upper Limb Movement Patterns in Children with Unilateral Cerebral Palsy. A Statistical Parametric Mapping Study
Source: Front Hum Neurosci. 2017 Oct 5;11:482. doi: 10.3389/fnhum.2017.00482 (PMC5633911; doi:10.3389/fnhum.2017.00482)
Supplement: Supplementary file 3 [file Image3.pdf]

Supplementary Material 3

Negative influence of motor impairments on upper limb movement patterns in children with unilateral cerebral palsy. A statistical parametric mapping study

Simon-Martinez, C*; Jaspers, E; Mailleux, L; Desloovere, K; Vanrenterghem, J; Ortibus, E; Molenaers, G; Feys, H; Klingels, K

*** Correspondence:** Cristina Simon-Martinez; cristina.simon@kuleuven.be

Impact of spasticity on UL movement patterns in children with uCP during the RF, RS, RGS, HTH and HTM tasks for all joint angles. Each column corresponds to a joint (from left to right: wrist, elbow, shoulder and trunk). The top image of each column is the SPM output of the vector field analysis (non-parametric Canonical Correlation Analysis test, except for the wrist, where a non-parametric linear regression was computed). For visualization purposes, kinematic data was grouped according to the level of motor impairments, i.e. spasticity total score: low impairments (score between 0 and 2, i.e. values above percentile 75), moderate impairments (score between 2.5 and 3.5, i.e. values between percentile 25 and 75), and severe impairments (score between 4 and 7, i.e. values below percentile 25). Below, mean (bold line) and standard deviation (translucent area) of the low impairments (green), moderate impairments (blue) and severe impairments (red) of each vector component and the respective post-hoc SPM{t} output. The black bar under each kinematic profile indicates clusters of significant influence of the impairment level on the kinematic variable.

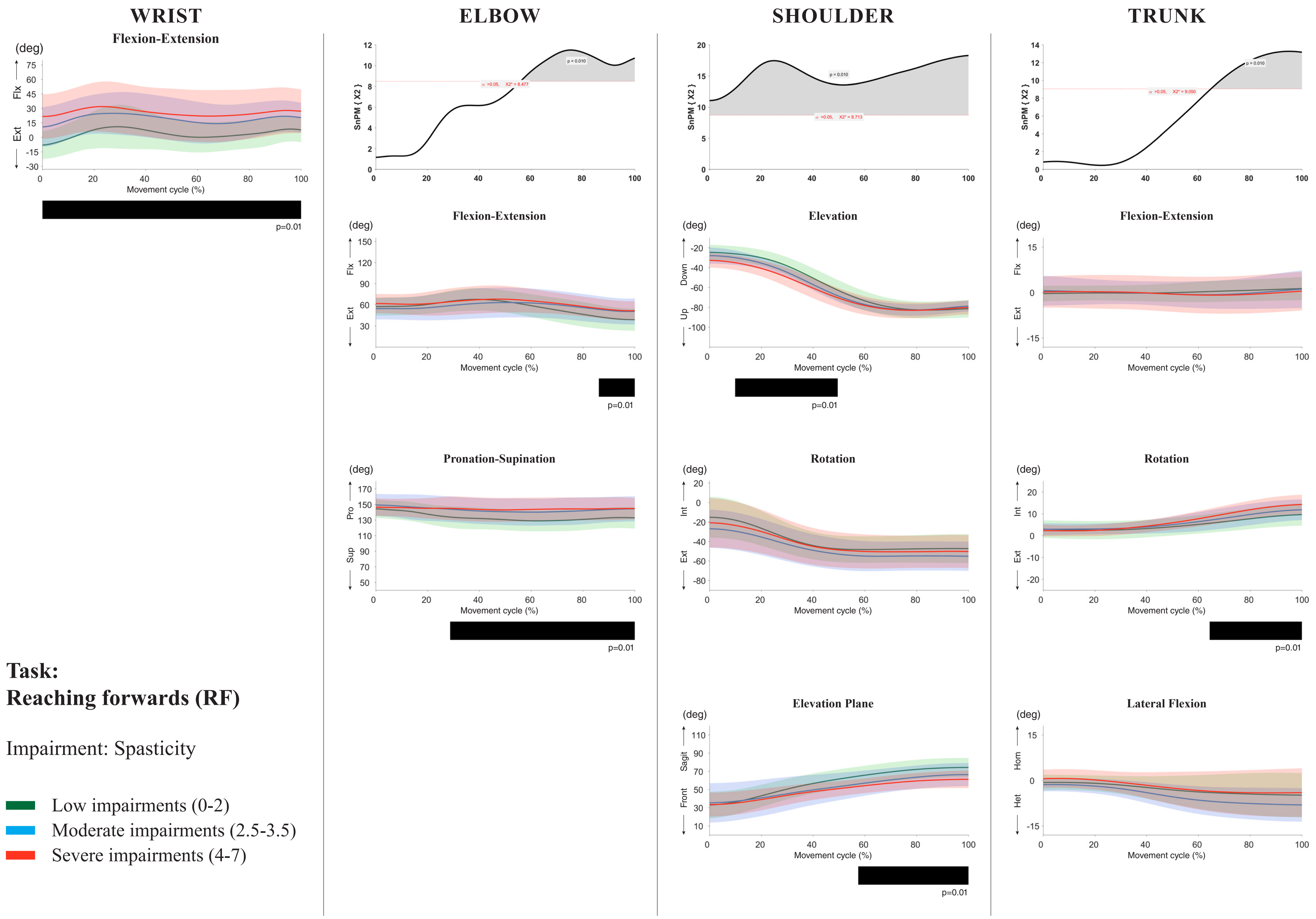
WRIST
ELBOW
SHOULDER
TRUNK
Flexion-Extension
(deg)
Flx
Ext
Movement cycle (%)
p=0.01
SnPM { X2 }
p = 0.010
Flexion-Extension
Elevation
Down
Up
Pronation-Supination
Pro
Sup
Rotation
Int
Ext
Elevation Plane
Sagit
Front
Lateral Flexion
Hom
Het
Task:
Reaching forwards (RF)
Impairment: Spasticity
Low impairments (0-2)
Moderate impairments (2.5-3.5)
Severe impairments (4-7)

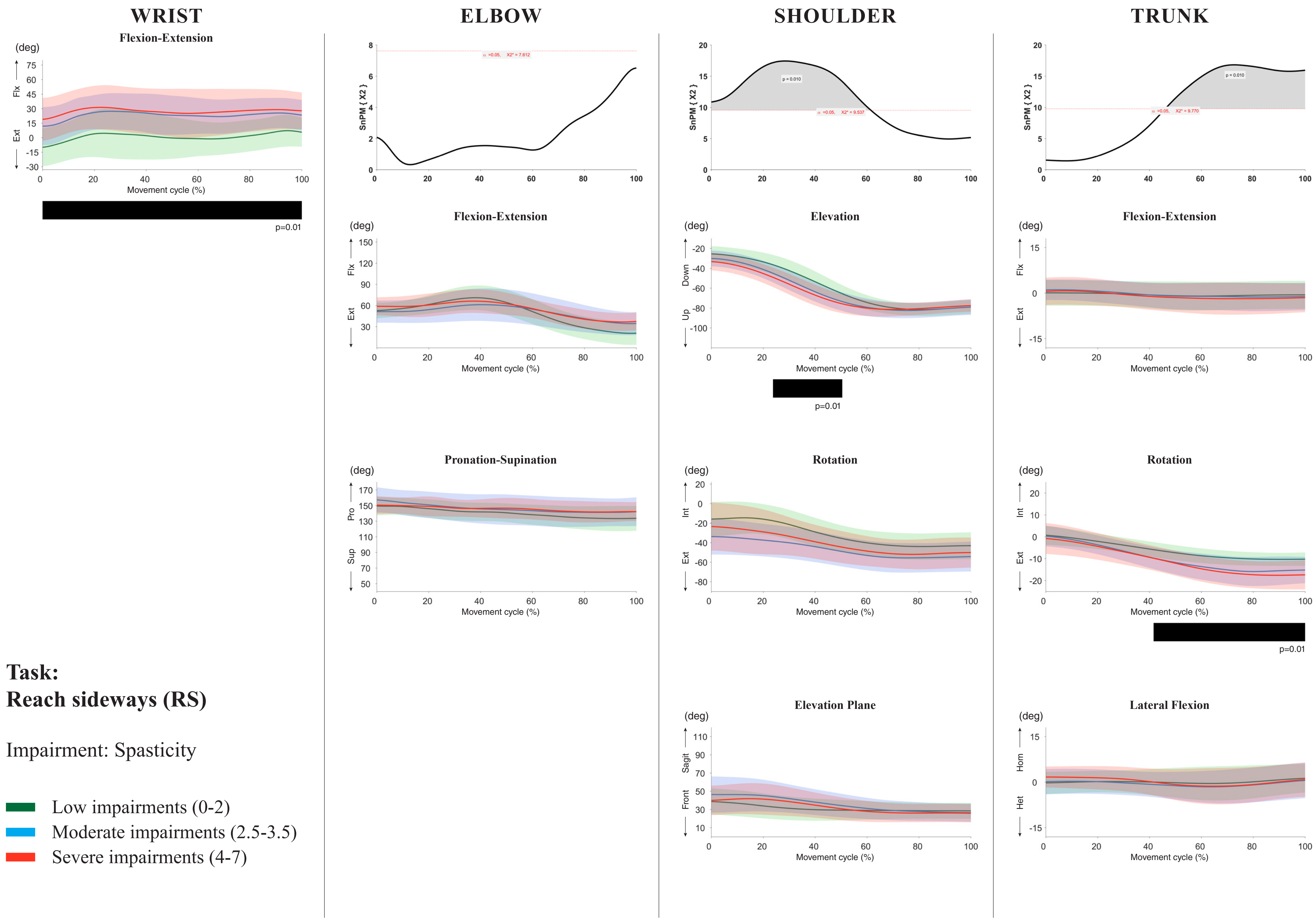

WRIST
ELBOW
SHOULDER
TRUNK
Flexion-Extension
Flexion-Extension
Pronation-Supination
Elevation
Rotation
Elevation Plane
Flexion-Extension
Rotation
Lateral Flexion
SnPM { X2 }
(deg)
Flx
Ext
Pro
Sup
Down
Up
Int
Ext
Sagit
Front
Hom
Het
Movement cycle (%)
p=0.01
p = 0.010
Task:
Reach sideways (RS)
Impairment: Spasticity
Low impairments (0-2)
Moderate impairments (2.5-3.5)
Severe impairments (4-7)

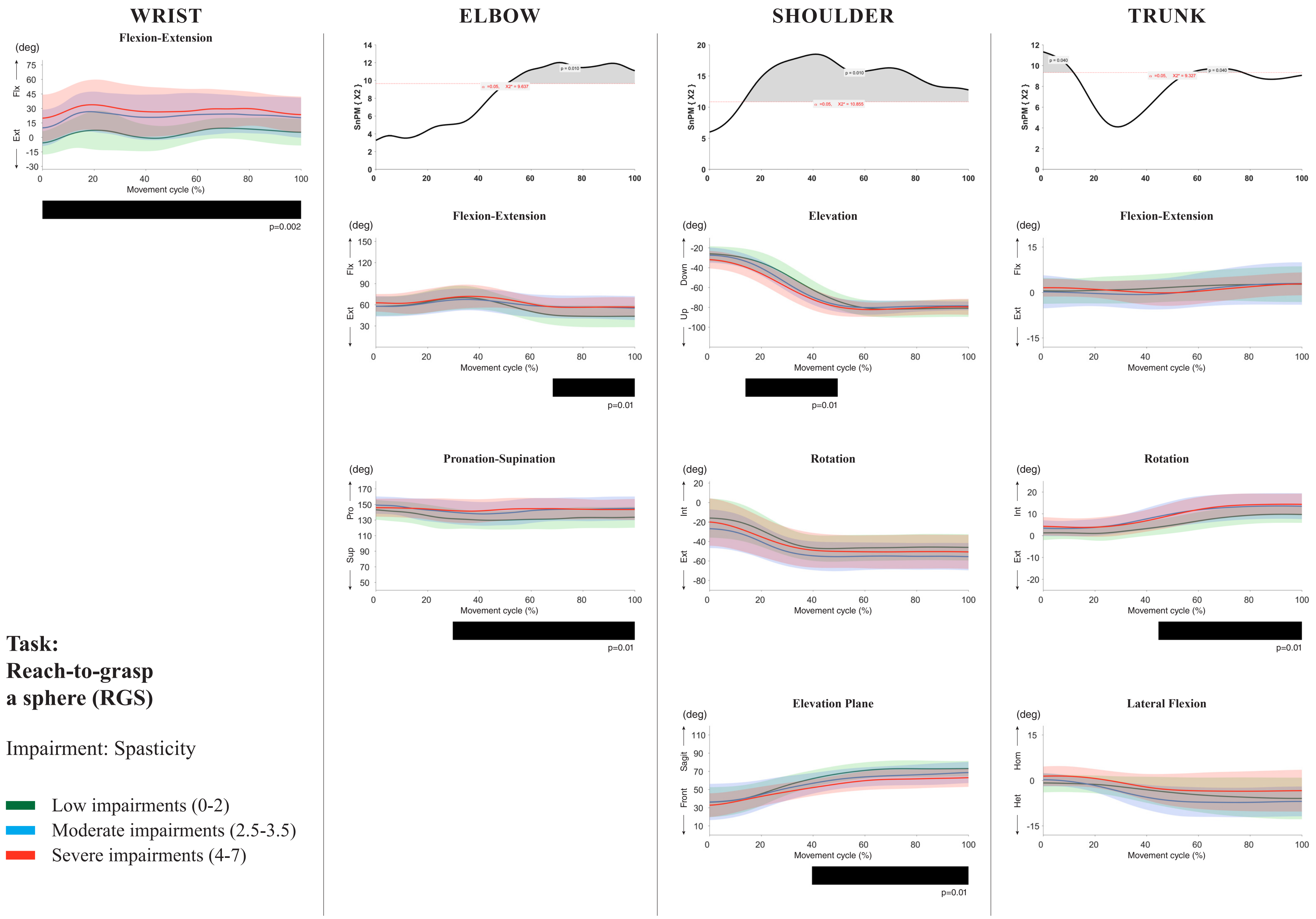
WRIST
ELBOW
SHOULDER
TRUNK
Flexion-Extension
(deg)
Flx
Ext
Movement cycle (%)
p=0.002
SnPM { X2 }
p = 0.010
α =0.05, X2* = 9.637
α =0.05, X2* = 10.855
p = 0.040
α =0.05, X2* = 9.327
Flexion-Extension
p=0.01
Elevation
Down
Up
p=0.01
Flexion-Extension
Pronation-Supination
Pro
Sup
p=0.01
Rotation
Int
Ext
Rotation
Int
Ext
p=0.01
Elevation Plane
Sagit
Front
p=0.01
Lateral Flexion
Hom
Het
Task:
Reach-to-grasp
a sphere (RGS)
Impairment: Spasticity
Low impairments (0-2)
Moderate impairments (2.5-3.5)
Severe impairments (4-7)

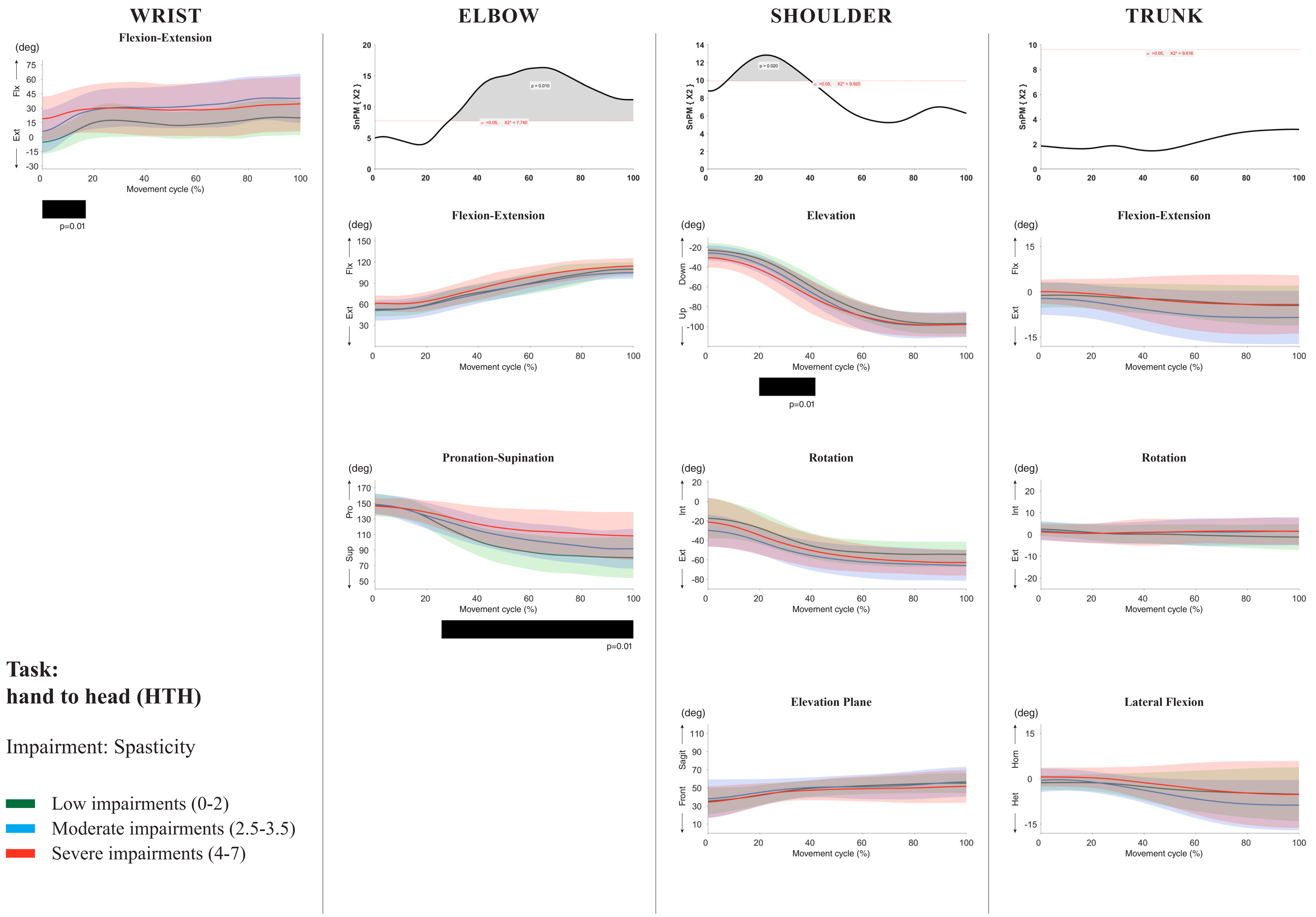
WRIST
ELBOW
SHOULDER
TRUNK
Flexion-Extension
(deg)
Flx
Ext
Movement cycle (%)
p=0.01
SnPM { X2 }
p = 0.010
p = 0.020
Flexion-Extension
Elevation
Down
Up
Pronation-Supination
Pro
Sup
Rotation
Int
Ext
Elevation Plane
Sagit
Front
Lateral Flexion
Hom
Het
Task:
hand to head (HTH)
Impairment: Spasticity
Low impairments (0-2)
Moderate impairments (2.5-3.5)
Severe impairments (4-7)

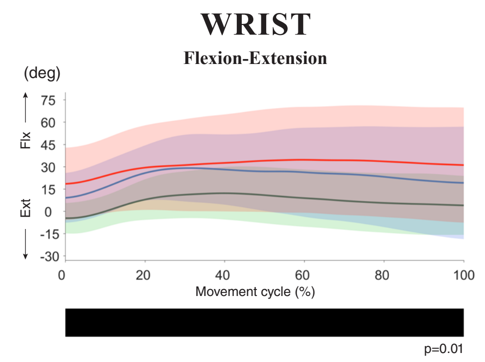

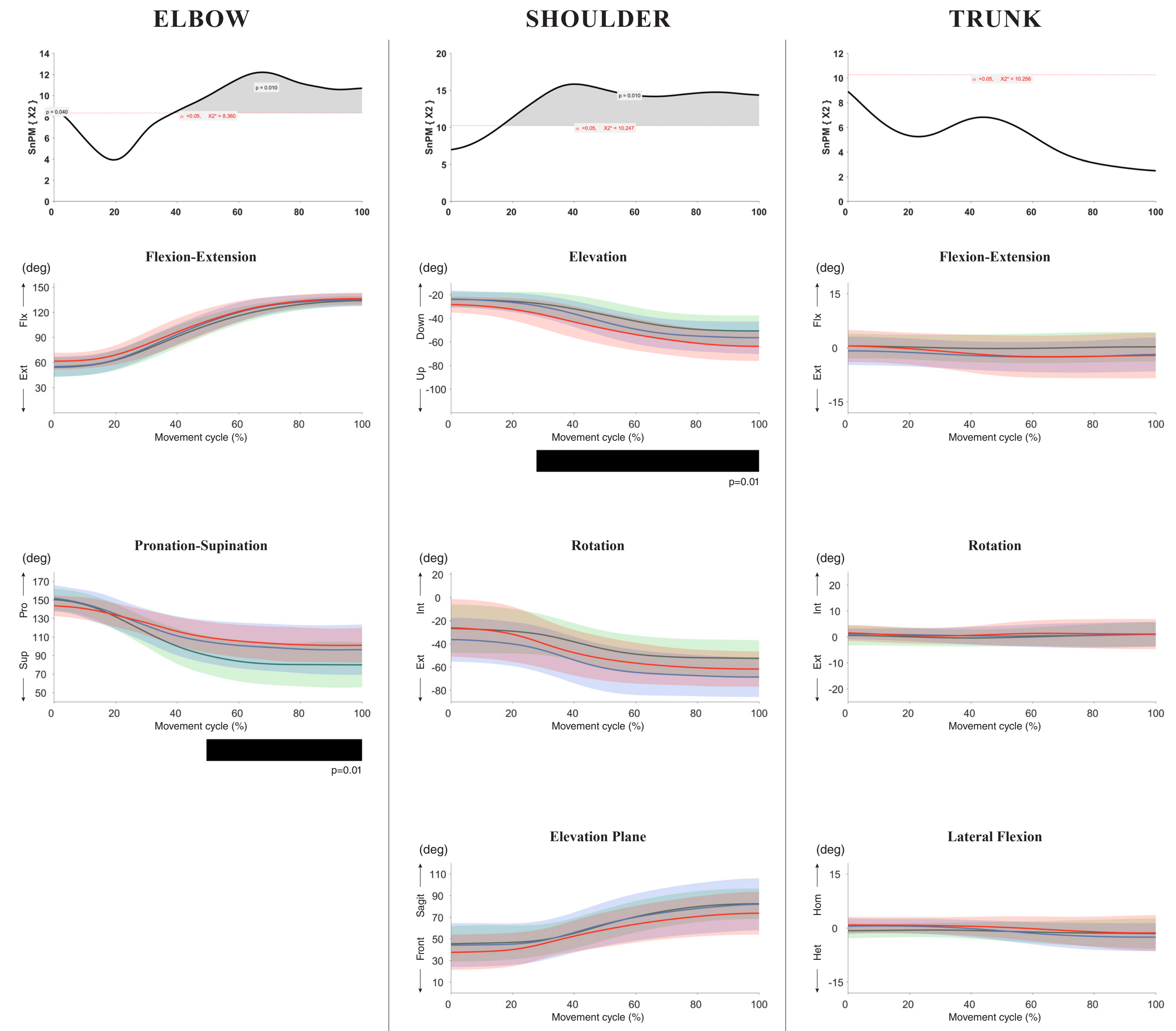

Task:
hand to mouth (HTM)

Impairment: Spasticity

Low impairments (0-2)
Moderate impairments (2.5-3.5)
Severe impairments (4-7)
